# Carbohydrate reserve partitioning and reproductive decline following defoliation-induced carbon source limitation in mango (*Mangifera indica*)

**DOI:** 10.1093/treephys/tpag069

**Published:** 2026-05-22

**Authors:** Gerhard C Rossouw, Bruno R Tamelini, Carole Wright, Sophie Jones, Geoffrey Dickinson, Ryan Orr

**Affiliations:** Queensland Department of Primary Industries, Mareeba Research Facility, Mareeba, Queensland 4880, Australia; Queensland Department of Primary Industries, Mareeba Research Facility, Mareeba, Queensland 4880, Australia; Queensland Department of Primary Industries, Mareeba Research Facility, Mareeba, Queensland 4880, Australia; School of Agriculture and Food Sustainability, The University of Queensland, Hartley Teakle Building, St Lucia, Queensland 4072, Australia; ARC Centre of Excellence for Plant Success in Nature and Agriculture, The University of Queensland, Mansfield Place, St Lucia, Queensland 4072, Australia; Queensland Department of Primary Industries, Mareeba Research Facility, Mareeba, Queensland 4880, Australia; Queensland Department of Primary Industries, Mareeba Research Facility, Mareeba, Queensland 4880, Australia

**Keywords:** carbon source–sink relationships, fruit productivity, horticulture, leaf area, non-structural carbohydrates, starch reserves

## Abstract

Non-structural carbohydrate (NSC) reserves can support fruit development and buffer source–sink imbalances in fruit trees, yet their organ-specific contributions remain poorly understood in mango (*Mangifera indica* L.). This study investigated the impacts of severe carbon source limitation on fruit productivity and NSC reserve dynamics over two consecutive growing seasons. A near-complete defoliation, retaining only 40 leaves (<0.5% of estimated leaves per tree), was imposed during rapid fruit growth in the first season. Starch and soluble sugar concentrations were monitored in roots, trunks, branches, shoots, pedicels and leaves, alongside fruit growth and yield. Fruit growth, number and dry matter content declined, with whole-tree fruit NSC content reduced to one-third of controls by end of the first season. In the following season, fruit NSC concentrations recovered to control levels, but reproductive output remained impaired, with fewer fruiting trees and lower yields, highlighting lasting impacts of prior source limitation on reproductive processes. Non-structural carbohydrate reserve dynamics varied across organs. Trunks behaved as long-term starch storage tissues, exhibiting marked depletion under defoliation in the first season, followed by strong recovery in the second. Coarse roots, assessed only in the second season, also showed similar storage behavior, with previously defoliated trees ending the season with higher reserve concentrations than controls. Branches and medium roots operated as more transient buffering tissues, with dynamics in medium roots driven more by phenology than tree carbon status. Shoots, leaves and pedicels, dominated by soluble sugars, served as short-term buffers, rapidly depleted under canopy loss but recovering once fruit load was reduced. Mango trees responded to source limitation through widespread NSC reserve remobilization, followed by synchronized reserve rebuilding across structural organs at the expense of reproduction. These findings emphasize the central role of foliar source capacity in sustaining productivity and clarify the hierarchical functions of NSC pools in buffering carbon limitation.

## Graphical Abstract

**Graphical Abstract:**
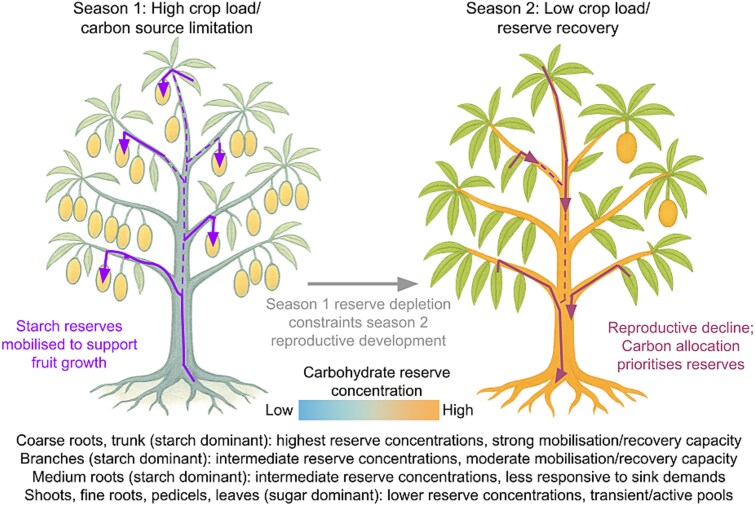

## Introduction

Erratic and alternate inter-seasonal bearing in mango (*Mangifera indica* L.) and other fruit crops remains a major challenge for commercial production, often linked to imbalances in non-structural carbohydrate (NSC) availability (Sharma et al. 2019, Capelli et al. 2021). Non-structural carbohydrate reserves, primarily starch and soluble sugars, buffer temporal mismatches between photosynthetic carbon supply and sink demand during key phenological stages such as flowering, fruit set and fruit growth (Lemoine et al. 2013, Rossouw et al. 2024*b*). This buffering capacity is increasingly recognized as a key determinant of productivity and resilience in trees, particularly under conditions of episodic carbon stress and strong reproductive demand (Campbell and Kalcsits 2024, Nawaz et al. 2025). However, organ-specific NSC dynamics over consecutive seasons under imbalanced source–sink conditions remain poorly characterized in mango. This limits mechanistic understanding of how whole-tree reserve security is maintained, depleted or compromised across seasons, an uncertainty highlighted more broadly in woody plants (Landhäusser and Adams 2024). This gap is particularly relevant given mango’s strong tendency for alternate bearing, increasingly attributed to NSC reserve depletion during heavy cropping years (Das et al. 2019). Without clearer insight into stress-induced reserve mobilization and replenishment, it remains difficult to anticipate carry-over effects on reproduction or to define informative tissues and sampling windows for diagnosing recovery.

In mango, starch reserves accumulate primarily in perennial woody organs such as roots, trunks and scaffold branches, acting as sinks when carbon is in surplus and as sources during high-demand periods (Davie and Stassen 1997, Rossouw et al. 2024*b*). This transition is particularly important during fruit development, when mobilized reserves supplement current photosynthesis to sustain reproductive growth (Chacko et al. 1982). Repeated or severe depletion of NSCs, whether from heavy fruit load or impaired photosynthesis, may constrain subsequent flowering and fruit set, contributing to yield instability (Sharma et al. 2019). While mango trees store substantial reserves in woody tissues (Davie et al. 1999, Rossouw et al. 2024*b*), the relative contribution of different organs to storage, mobilization and recovery remains poorly resolved, especially under acute carbon source stress. Recent work in other tree species suggests that organ-level differences in reserve depletion and replenishment become most apparent under severe carbon limitation (Zahnd et al. 2024) and that seasonal dynamics in NSC fluctuations are expressed in an organ-specific manner (Guo et al. 2025). Such conditions therefore provide a critical window for identifying which organs buffer carbon stress and which incur longer-term costs. This information is essential for understanding whole-tree buffering capacity, the physiological constraints governing yield consistency across seasons, and for guiding effective sampling strategies to assess reserve status and recovery.

Starch and soluble sugars (predominantly sucrose, glucose and fructose) differ in turnover rate, mobility and functional role within the plant’s carbon economy (Sala et al. 2012, Long and Adams 2022). Starch acts as a long-term carbon reserve, while soluble sugars are readily phloem transportable. In leaves, photosynthate is temporarily stored as starch and remobilized overnight to sustain sink demand (Lemoine et al. 2013). In contrast to these short-term foliar dynamics, NSC reserves in woody organs buffer longer-term carbon deficits, particularly during reproductive development or under stress-induced source limitation (Loescher et al. 1990, Rossouw et al. 2024*b*). Storage capacity also varies among organs, with trunks and coarser roots typically storing greater absolute quantities than smaller branches or fine roots (Stassen and Janse van Vuuren 1997). Spatial mobilization patterns may vary, with proximal tissues (for example, shoots) potentially being accessed first due to vascular proximity, while distal tissues such as roots and trunks may be mobilized more slowly (Hart et al. 2024, Rossouw et al. 2024*a*). These spatial gradients in reserve use remain underexplored in mango, limiting our ability to predict how different tissues contribute to whole-tree reserve recovery following carbon stress and associated source–sink imbalances.

Although starch reserve depletion in mango can result from drought, shading or heavy fruit load (Chacko et al. 1982, Léchaudel and Joas 2006), artificial defoliation provides a controlled method to impose acute carbon limitation and evaluate compensatory responses (Rossouw et al. 2017*a*, Wiley et al. 2017). When applied during rapid fruit growth, a period of already high sink demand, defoliation creates a carbon-deficit scenario that mimics commercial stress conditions and enables detailed analysis of source–sink trade-offs and recovery dynamics. Defoliation has been used to investigate source–sink dynamics in other fruiting species such as apple (*Malus domestica*), grapevine (*Vitis vinifera*) and kiwifruit (*Actinidia deliciosa*) (Cruz-Castillo et al. 2010, Rossouw et al. 2017*a*, 2024*a*). Beyond defoliation-based studies, evidence from tree crops including apple, pistachio (*Pistacia vera*) and olive (*Olea europaea*) shows that NSC-mediated buffering plays a central role in sustaining yield stability, although species differ in their reserve storage strategies, organ-level sink priorities and recovery trajectories (Spann et al. 2008, Naschitz et al. 2010, Bustan et al. 2011). At a broader physiological scale, NSCs are key integrators of stress resilience in woody plants, contributing to recovery and buffering against abiotic stressors including carbon limitation (Nawaz et al. 2025), while organ-level storage and allocation strategies vary systematically among tissues and species (Zhang et al. 2024). Leaf-to-fruit ratio manipulation in mango has been shown to influence leaf photosynthesis, fruit growth and dry matter accumulation (Urban and Léchaudel 2005, Léchaudel and Joas 2006). However, the specific effects of acute leaf area loss on NSC reserve dynamics, tissue-level recovery and subsequent reproductive performance remain poorly resolved. This knowledge gap limits mechanistic understanding and effective management strategies under severe, transient carbon-limiting conditions (Loescher et al. 1990, Sala et al. 2012, Landhäusser et al. 2018).

Here, we investigate how severe carbon source limitation during fruit development alters the mobilization and subsequent replenishment of NSC reserves across the whole mango tree, using a near-complete defoliation treatment that retained only 40 mature leaves per tree from canopies estimated to normally bear several thousand leaves (Wu et al. 2018). Specifically, we test whether acute leaf area loss forces differential mobilization of NSC reserves among organs, whether priority is given to sustaining fruit growth at the expense of reserve security, and the extent to which depletion of long-term storage pools constrains reproductive capacity in the following season. By quantifying NSCs in roots (separated by diameter class), trunks, scaffold branches, vegetative and reproductive shoots, fruit-bearing panicles, developing fruit and leaves, we provide a high-resolution, whole-tree perspective on mango carbon allocation under carbon stress and recovery. These insights strengthen mechanistic understanding of NSC partitioning and buffering in mango and offer a framework for interpreting carbon-mediated yield instability in other perennial fruit systems.

## Materials and methods

### Site and planting material

This study was conducted over two consecutive growing seasons (2023–24 and 2024–25) at the Queensland Department of Primary Industries’ Walkamin Research Facility (17°07′ S, 145°25′ E), located in a tropical savannah climate (Köppen Aw). The region has a dry season from May to September, with a 10-year mean monthly rainfall of 19 mm across this period. There is a pronounced wet season from December to March, with a 10-year mean monthly rainfall of 234 mm across these months, and transitional months in October–November and April. Climate data were obtained from the Australian Bureau of Meteorology Walkamin weather station (station number 031108). Meteorological conditions during the 2023–24 data collection period, including daily rainfall, minimum and maximum air temperatures, and solar radiation, are provided (see Figures S2, S3 and S4 available as Supplementary Data at *Tree Physiology* Online). The experiment used ‘Keitt’ mango trees grafted onto ‘Kensington Pride’ rootstock, established in December 2013 at 6 × 4 m spacing (row × tree) as part of a long-term planting systems trial (Mahmud et al. 2023, Ibell et al. 2024). The defoliation treatment, as described below, was timed to coincide with fruit growth during the wet season, when carbon demand is high and vegetative flushing is minimal, to maximize the imposed carbon source limitation. Trees were trained to a central leader system and managed according to commercial standards for irrigation, fertilization, pest and disease control, and annual postharvest pruning (Meurant et al. 1999). Tree rows were oriented north–south.

### Experimental design and defoliation treatment

Twelve trees across four orchard rows were randomly allocated into six control–treatment pairs. Edge trees were excluded, and buffer trees separated each pair. Each individual tree constituted one biological replicate (*n* = 6 per treatment), and the same trees were sampled repeatedly at each sampling date across the study period. On 6 December 2023, during the rapid fruit expansion phase (BBCH 703–705), a near-complete defoliation treatment was imposed to simulate severe carbon source limitation, ~96 days after anthesis. The BBCH scale (Biologische Bundesanstalt, Bundessortenamt und Chemische Industrie), referred to henceforth, provides a standardized system for describing phenological development in mango (Hernández Delgado et al. 2011).

In each defoliated tree, only 40 mature leaves were retained (10 per cardinal direction, distributed evenly across the canopy), all located on apical shoot parts adjacent to fruit-bearing panicles. These leaves were retained to enable assessment of leaf functional responses in a companion study and to track potential compensatory foliar NSC dynamics. However, their contribution to overall canopy assimilation was considered negligible. Based on published estimates of whole-tree and individual leaf area in mango (Chacko et al. 1982, Wu et al. 2018), retaining only 40 leaves was estimated to represent <0.5% of the expected canopy leaf number, equivalent to removal of >99% of leaves. Total canopy leaf number or leaf area was not directly quantified in the current study, so this estimate should be interpreted as an approximate indicator of treatment severity. This assumption is consistent with reports from mango and other fruit trees, where substantial reductions in leaf area markedly reduce whole-tree carbon assimilation and carbon supply to sinks (Chacko et al. 1982, Léchaudel and Joas 2006, Rossouw et al. 2018). Control trees were left fully foliated. New vegetative growth in treated trees was removed fortnightly to maintain source limitation until harvest. Specifically, all newly emerged vegetative flushes were removed at an early developmental stage, typically when leaf tips were first visible (<5 cm) above bud scales and prior to substantial leaf unfolding or expansion. Entire flush shoots were excised at their point of emergence, preventing the development of new photosynthetically active leaf area.

In the second season (2024–25), all trees were pruned within 2 weeks after harvest of the first season in accordance with standard commercial practice, involving mechanical hedging of shoot tips followed by selective removal of dead or overlapping branches. No defoliation or vegetative removal treatments were imposed. Trees were allowed to develop postharvest vegetative flushes and proceed through flowering and fruit development under unaltered canopy conditions.

### Fruit growth and yield

At treatment imposition (6 December 2023), 10 fruit per tree were tagged (five representative fruit per east and west canopy side). Fruit dimensions (length, width and depth) were measured on seven occasions during the rapid fruit growth period: 6 December, 11 December, 20 December in 2023 and 4 January, 15 January, 24 January and 7 February in 2024. These measurement dates were selected to capture fruit growth dynamics during the period of rapid cell expansion following treatment imposition, with approximately weekly to fortnightly intervals maintained where possible given weather conditions during the wet season. Fruit volume was estimated as an ellipsoid [(π/6) × (length × width × depth)]. Mean daily growth rates were calculated from successive volume increments.

Fruit were harvested at physiological maturity (BBCH 800), assessed by skin color change in control trees and by ≥12% flesh dry matter, on 8 February 2024 (first season) and 3 February 2025 (second season). All fruit were counted and weighed to determine yield per tree, and average fruit fresh mass was calculated. A subsample of 25 fruit per tree was used to estimate tree-level dry matter. Flesh samples were collected with paired 6 cm × 2 cm cores from opposing sides at the midsection. Samples were peeled, pooled per fruit, weighed fresh and oven-dried at 60 °C to constant mass to determine dry matter percentage. This percentage was then applied to total fresh yield to calculate dry yield, and subsequently used to calculate whole-tree fruit NSC content at harvest, as described later.

### Non-structural carbohydrate sampling framework

Non-structural carbohydrate dynamics were assessed in fruit and multiple vegetative and structural organs to characterize whole-tree carbon allocation during acute carbon limitation and subsequent recovery. Sampling dates for each season are summarized in Table 1. Across the two seasons, the following tissues were sampled: fruit flesh; fine, medium and coarse roots; trunk wood; primary and tertiary scaffold branches; reproductive and vegetative apical shoot terminals; and fruit-bearing pedicels. Leaves, medium roots, trunks, shoot terminals and fruit pedicels were sampled in both seasons, while fine and coarse roots, and branches were sampled only during the 2024–25 season. All tissues were sampled from the same individual trees at each appropriate sampling date, with each tree representing one biological replicate (*n* = 6 per treatment).

**Table 1:** Sampling schedule for non-structural carbohydrate (NSC) analysis across two consecutive seasons (2023–24 and 2024–25). Collection times are given as days after defoliation treatment, with reference to anthesis and phenological stages shown in a separate column.

Collection time (days after defoliation)	Reference to anthesis and phenology	Tissues sampled
2023–24		
1	97 days after anthesis; rapid fruit growth	Fruit flesh, medium roots, trunks, fruiting shoot terminals, fruit pedicels, leaves
15	111 days after anthesis; rapid fruit growth	Fruit flesh, medium roots, trunks, fruiting shoot terminals, fruit pedicels, leaves
36	132 days after anthesis; rapid fruit growth	Fruit flesh, medium roots, trunks, fruiting shoot terminals, fruit pedicels, leaves
64	160 days after anthesis; fruit harvest	Fruit flesh, medium roots, trunks, fruiting shoot terminals, non-fruiting shoot terminals, fruit pedicels, leaves
2024–25		
128	145 days before anthesis; vegetative development	Medium roots, fine roots, coarse roots, trunks, primary branches, tertiary branches, leaves
222	51 days before anthesis; panicle emergence	Medium roots, fine roots, coarse roots, trunks, primary branches, tertiary branches, leaves
309	36 days after anthesis; early fruit growth	Fruit flesh, medium roots, fine roots, coarse roots, trunks, primary branches, tertiary branches, fruiting shoot terminals, non-fruiting shoot terminals, leaves
442	149 days after anthesis; fruit harvest	Fruit flesh, medium roots, fine roots, coarse roots, trunks, primary branches, tertiary branches, fruiting shoot terminals, non-fruiting shoot terminals, fruit pedicels, leaves

The inter-seasonal tissue sampling strategy reflected logistical and temporal constraints during the first season, which represented a single late-season phenological window and required prioritization of core tissues. In contrast, the longer sampling intervals across the full second season allowed inclusion of additional organs without reducing sampling intensity or replication. The sampling intervals varied among tissue types and are outlined in Table 1 for each season. All samples were snap-frozen in liquid nitrogen immediately after collection, transported on dry ice and stored at −80 °C until processing.

In the 2023–24 season, sampling occurred on 7 December (1 day after defoliation), 21 December, 11 January and 8 February (harvest). In the 2024–25 season, sampling occurred on 12 April (vegetative flush maturity; BBCH 319), 15 July (early panicle emergence; BBCH 513), 10 October (early fruit growth; BBCH 701) and 31 January (fruit maturity; BBCH 800). For each tissue and sampling date, one composite sample per tree was analyzed, maintaining tree-level biological replication across all tissues.

Fruit sampling involved collection of five representative fruit per tree at each applicable sampling date, pooled to generate one composite fruit flesh sample. Fruit flesh was cored from both sides and pooled. At harvest, an additional 15 fruit per tree from the dry matter determination cohort were processed for NSC analysis to allow calculation of whole-tree fruit NSC content (dry yield × concentration). For these fruits, a core was taken from the blushed side of each fruit and pooled per tree.

Root tissues were classified by diameter at collection: fine roots (~1 mm), medium roots (3–5 mm) and coarse roots (>10 cm diameter structural roots). Fine and medium roots were excavated, washed with distilled water to remove debris, trimmed of senescent tissue and blotted dry before being snap-frozen. Coarse roots were exposed, rinsed with distilled water, and cored to a depth of 5 cm using a 3.7 mm drill bit; two cores per tree were pooled. Trunk wood was sampled by drilling and combining two cores per tree, 5–15 cm above the graft union. Sampling positions were aligned with the rotated root quadrants to ensure representative coverage. Branch wood samples were collected from two structural positions to assess NSC distribution along the canopy framework: (i) near the apex of the primary branch (i.e. the single leader), and (ii) from tertiary scaffold branches, defined as second-order branches extending from the main (secondary) scaffold limbs. For the leader branch, cores were taken by drilling near the apical region using the same four quadrant orientations as those used for trunks. For tertiary scaffold branches, one core was taken from each of three branches per tree and pooled to obtain a representative sample.

Shoot terminals were sampled by collecting 2–3 cm of apical internode tissue from shoots adjacent to fruiting or flowering panicles. Non-fruiting vegetative shoot internodes were collected near the shoot apex to assess reserve status in vegetative shoots. For both shoot types, five terminals per tree were collected and pooled. Fruiting pedicels were collected from the same fruit used for fruit flesh sampling. Each pedicel sample included the tissue just above and below the abscission zone (~3 mm above and 3 mm below, inclusive of the abscission zone). Five pedicels per tree were pooled. Leaf NSCs were assessed by collecting five interveinal discs (1 cm diameter) from 10 mature, healthy leaves per tree, sampled near shoot apices and where possible, adjacent to fruit-bearing panicles at each sampling date. Fifty discs were pooled per tree.

### Non-structural carbohydrate extraction, quantification and derived variables

Frozen fruit core samples were ground under liquid nitrogen using an A11 basic analytical mill (IKA, Selangor, Malaysia), freeze-dried (BK-FD18 series, Biobase, Shandong, China) and further homogenized to a fine powder using a bead mill (TissueLyser II, Qiagen, Clayton, Australia). Medium and fine root samples, as well as shoot terminals, were first freeze-dried, then initially ground using a benchtop hammer mill (DFH 48, Culatti, Glen Creston, Stanmore, UK), followed by fine milling using a bead mill. Coarse root, trunk, primary and tertiary branch, and leaf samples were freeze-dried and subsequently ground to a fine powder using a bead mill. Frozen pedicel samples were ground to a fine powder using pre-chilled stainless-steel grinding jars and balls in a bead mill, then freeze-dried prior to analysis.

Subsamples (20 mg) of freeze-dried powder were used to enzymatically quantify starch, sucrose, glucose and fructose concentrations with assay kits (K-TSTA and K-SUFRG, Megazyme International, Bray, Ireland), following the methods described in Rossouw et al. (2024a) and citations therein. Analytical precision was monitored using routine internal quality-control samples, analyzed in triplicate within each assay run. Briefly, soluble sugars were extracted using three sequential washes of 1 mL 80% (v/v) aqueous ethanol for 10 min each at 80 °C, with centrifugation between washes. Supernatants were combined, diluted to 10 mL with deionized water, and sucrose, glucose and fructose concentrations were quantified enzymatically using the K-SUFRG assay. In this method, each sugar was converted to glucose-6-phosphate (G6P), and reduced nicotinamide-adenine dinucleotide phosphate (NADPH) formation was quantified spectrophotometrically following oxidation in the presence of NADP^+^ and glucose-6-phosphate dehydrogenase. For leaves, sugar extracts were first decolorized using activated charcoal to remove potential chlorophyll interference in absorbance readings (Dayer et al. 2016). Total soluble sugars were calculated as the sum of sucrose, glucose and fructose. Total hexoses were defined as the sum of glucose and fructose, and sucrose:hexose ratios were derived for each tissue and sampling date. Means and standard errors for individual sugar concentrations (sucrose, glucose and fructose) are additionally provided (see Table S1 available as Supplementary Data at *Tree Physiology* Online).

For starch analysis, the remaining pellet after sugar extraction was resuspended in 200 μL dimethyl sulfoxide and heated at 98 °C for 10 min to solubilize starch. Starch was then enzymatically hydrolyzed using the K-TSTA assay, involving incubation with thermostable α-amylase in MOPS buffer at 98 °C for 15 min, followed by further hydrolysis with amyloglucosidase in sodium acetate buffer at 50 °C for 60 min. After centrifugation and dilution where required, glucose released from starch was quantified colorimetrically and starch concentration calculated accordingly.

For all samples, total NSC was defined as the sum of total soluble sugars and starch. For the additional fruit samples taken at harvest (from the dry matter subsample), NSC concentrations were combined with total dry yield per tree to estimate whole-tree fruit starch, total soluble sugar and total NSC content. To evaluate functional linkages in NSC dynamics among organs and provide an integrative, whole-tree perspective, changes in total NSC concentration (ΔNSC) were calculated for each tissue as the difference between the final and first sampling dates within each season. Only tissues sampled consistently across each season were included to ensure comparability among organs. Five organs were included in the first season (medium roots, trunk wood, reproductive shoot terminals, fruit pedicels and leaves) and seven in the second season (medium, fine and coarse roots, trunk wood, primary and tertiary branches and leaves).

### Statistical analysis

Linear mixed models (LMM) were fitted to compare the control and defoliated treatments for all continuous variables. For the continuous harvest measurements, a factor representing the treatment was fitted as the fixed effect and the random effects comprised a nested structure of replicate and tree within replicate. Fruit count at harvest was analyzed using a generalized linear model (GLM) assuming a negative binomial distribution and log-ratio link function. Terms representing replicate and treatment (control and defoliated) were fitted in the GLM. For the seasonal dynamic analyses, the main effects and interaction of treatment and time were fitted as the fixed effects in an LMM. A quadratic regression model was fitted to the fruit growth rate over time.

For all measurements, the two seasons were analyzed separately and significance testing was performed at the 0.05 level. Where a significant treatment × sampling date interaction was detected, results were interpreted on the basis of the interaction, and main effects were not discussed independently. The main effects of treatment and sampling date were only discussed if the interaction was not significant. Pairwise comparisons of significant effects were performed using Fisher’s protected 95% least significant difference (LSD). Transformations were applied in the LMM if the assumptions of normality or homogeneity of variance were not satisfied.

To assess functional linkages in NSC reserves among organs at the whole-tree level, Spearman rank correlation analyses were performed on seasonal changes in total NSC concentration (ΔNSC) among tissues within each season. Correlations were calculated separately for each season using only organs sampled consistently across that season. Correlation coefficients and associated *P*-values were used to assess patterns of coordinated variation in reserve depletion and replenishment among organs. All analyses were performed in Genstat 24th edition statistical software (VSN International, Hemel Hampstead, UK).

## Results

### Reproductive performance and fruit NSC dynamics

**Figure 1:**
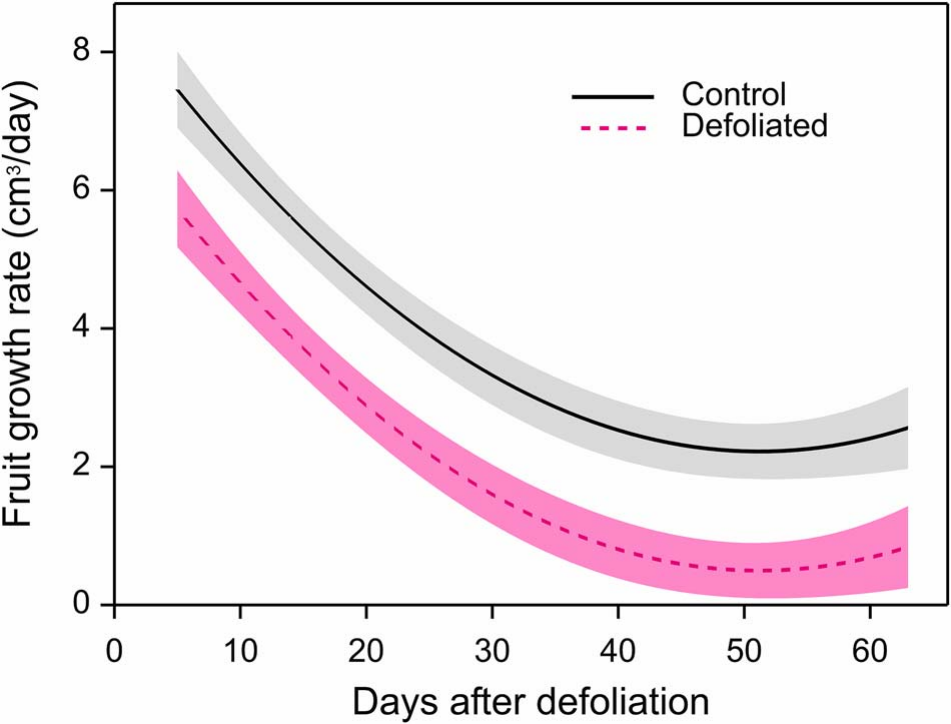
Quadratic regression curves of fruit growth rate in control (solid line) and defoliated (dashed line) mango trees following near-complete canopy removal in the 2023–24 growing season. Shaded areas represent 95% confidence intervals.

Fruit on defoliated trees exhibited consistently reduced growth rates across the 63 days from defoliation to harvest (Figure 1). A quadratic parallel lines model (adjusted R^2^ = 0.82) suggested a constant intercept offset of −3.3 cm^3^, indicating an estimated mean reduction of 3.3 cm^3^ day^−1^ volume gain compared with controls. Across both treatments, fruit growth rates declined gradually until ~ 40 days after defoliation, after which they stabilized.

In 2023–24, defoliation significantly reduced mean fruit number and yield per tree, and average fruit weight relative to controls (Table 2). Dry yield was approximately three fold lower reflecting fewer fruit, smaller size and reduced dry matter. Mean fruit starch concentration was approximately five fold lower in defoliated trees, resulting in more than a 16-fold reduction in total fruit starch content per tree. Although fruit from defoliated trees had higher mean sugar concentrations, total sugar content per tree was reduced by half. Overall, total NSC content in fruit was reduced to less than one-third of that in controls.

**Table 2:** Fruit number, yield, average fruit weight, dry matter content, dry yield and non-structural carbohydrate (NSC) concentrations and contents (starch, soluble sugars and total NSC per tree) at harvest across two consecutive seasons (2023–24 and 2024–25). Values are means. Standard errors (SE) and *P*-values are provided for each parameter within each season; for certain variables (e.g. fruit number), SE are reported separately for each treatment (control and defoliated) in line with the statistical analysis. Within rows, significant differences between treatments are indicated by *P* < 0.05.

	Control	Defoliation	*(SE)*	*P*
2023–24				
Fruits/tree	186.7	148.0		<0.001
*(SE)*	*(9.01)*	*(7.48)*		
Yield (kg/tree)	68.5	35.6	*(3.24)*	<0.001
Average weight (g/fruit)	367.2	241.8	*(7.96)*	<0.001
Dry matter (%)	12.6	8.5	*(0.13)*	<0.001
Dry Yield (kg/tree)	8.6	3.0	*(0.38)*	<0.001
Starch concentration (%)	38.1	8.0	*(1.14)*	<0.001
Total starch content (kg/tree)	3.7	0.2	*(0.06)*	<0.001
Total sugar concentration (%)	30.6	43.7	*(1.31)*	<0.001
Total sugar content (kg/tree)	2.6	1.3	*(0.14)*	<0.001
Total NSC concentration (%)	68.7	51.7	*(0.67)*	<0.001
Total NSC content (kg/tree)	5.9	1.6	*(0.25)*	<0.001
2024–25				
Fruits/tree	116.3	36.6		0.178
*(SE)*	*(73.48)*	*(20.37)*		
Yield (kg/tree)	63.3	24.2	*(9.46)*	0.004
Average weight (g/fruit)	583.0	730.5		0.098
*(SE)*	*(56.77)*	*(66.35)*		
Dry matter (%)	12.5	13.7		0.008
*(SE)*	*(0.31)*	*(0.32)*		
Dry Yield (kg/tree)	7.9	3.3	*(1.28)*	0.010
Starch concentration (%)	44.2	46.4		0.014
*(SE)*	*(0.66)*	*(0.68)*		
Total starch content (kg/tree)	3.5	2.8		0.233
*(SE)*	*(0.36)*	*(0.47)*		
Total sugar concentration (%)	33.6	34.6		0.351
*(SE)*	*(0.65)*	*(0.87)*		
Total sugar content (kg/tree)	2.7	2.0		0.233
*(SE)*	*(0.30)*	*(0.38)*		
Total NSC concentration (%)	77.8	80.7		0.072
*(SE)*	*(0.41)*	*(0.71)*		
Total NSC content (kg/tree)	6.1	4.8		0.207
*(SE)*	*(0.66)*	*(0.85)*		

In 2024–25, carryover effects of prior defoliation were evident (Table 2). Fruit number was lower in previously defoliated trees, although the difference was not statistically significant. Fresh yield was reduced by ˃60%, and dry yield was more than halved. Mean fruit mass tended to be higher than controls but did not differ significantly. Fruit dry matter and starch concentrations were higher in previously defoliated trees, while sugar and total NSC concentrations were comparable to controls. Despite these compositional differences, total starch, sugar and NSC contents per tree were consistently higher in controls, although not significantly so due to high variability among previously defoliated trees. Only three of six previously defoliated trees produced fruit in the second season compared with all six controls, contributing to reduced group means and increased variance.

**Figure 2:**
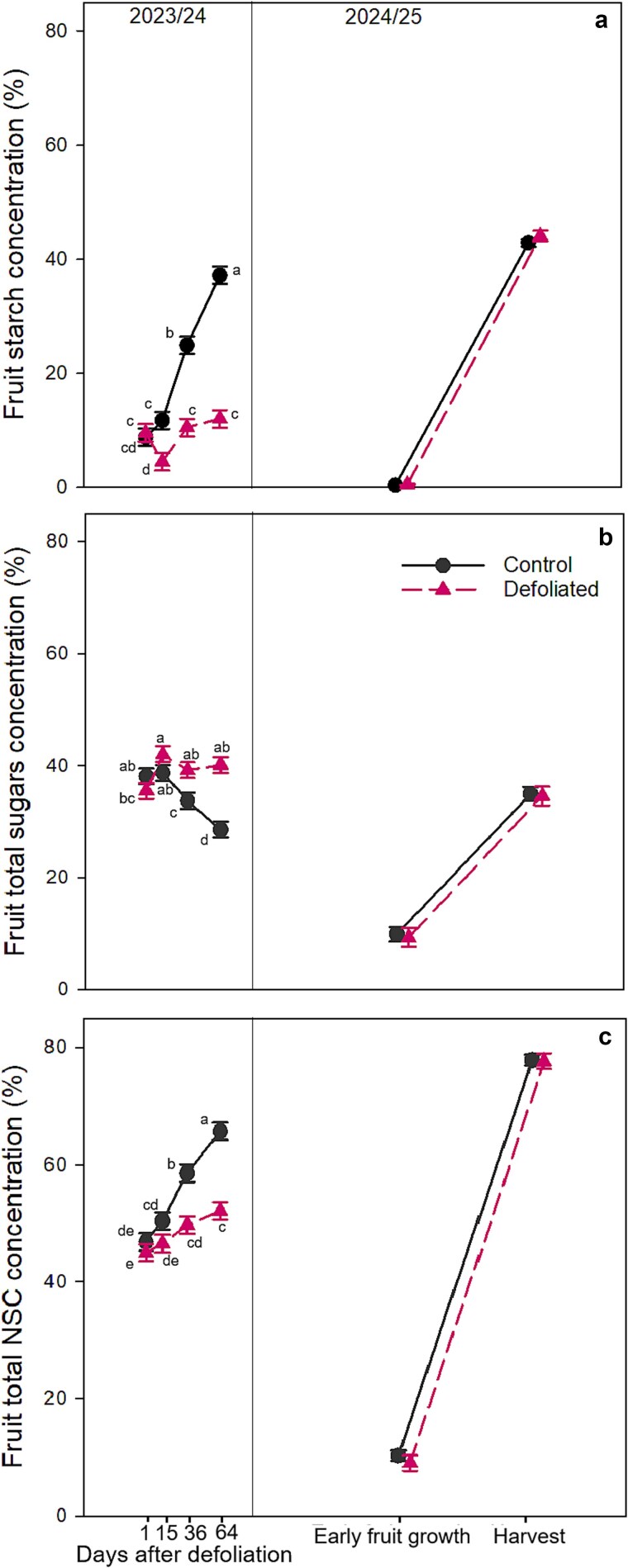
Seasonal dynamics of fruit non-structural carbohydrate (NSC) concentrations in control and defoliated trees during the 2023–24 and 2024–25 seasons. (a) Shows starch, (b) soluble sugars and (c) total NSC concentrations. Different lowercase letters within each season indicate statistically significant differences for the treatment × sampling time interaction (*P* < 0.05). Error bars represent ±1 standard error of the mean.

In 2023–24, fruit starch accumulation diverged between treatments, with a significant treatment × date interaction (*P* < 0.001; Figure 2a). Within 15 days after defoliation, starch declined in fruit from defoliated trees while rising in controls, creating a widening gap. By day 36, starch concentrations in controls were more than twice those in fruit from defoliated trees, and by harvest (day 64) the difference had tripled. Fruit total sugar concentrations also showed a significant treatment × date interaction (*P* < 0.001; Figure 2b). In controls, sugar concentrations declined gradually, while in defoliated trees they rose initially then remained stable. By harvest, fruit from defoliated trees contained significantly higher sugar concentrations than controls. Total NSC concentrations largely mirrored starch trends, with a significant interaction of treatment and date (*P* = 0.003; Figure 2c).

**Figure 3:**
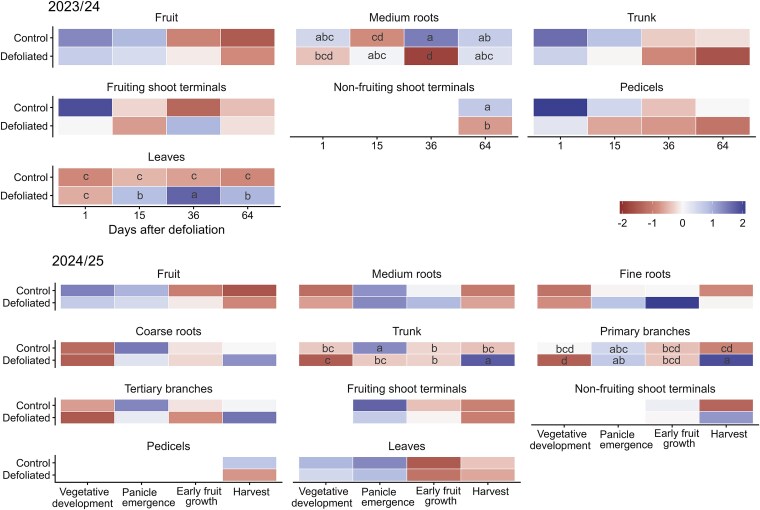
Heatmaps showing Z-score standardized sucrose:hexose ratios across organs in control and defoliated trees during the 2023–24 and 2024–25 seasons. Values were standardized within each organ as deviations from the organ mean divided by the corresponding standard deviation. Rows represent treatments and columns represent sampling dates. The heatmap scale indicates the relative deviation from the organ mean (Z-score), with positive values representing values above the organ mean and negative values representing values below the organ mean. Lowercase letters denote statistically significant treatment × sampling time interactions (*P* < 0.05).

In 2024–25, no significant effects of prior defoliation were detected for fruit starch, sugar or total NSC concentrations (*P* > 0.05; Figure 2). For both treatments, starch concentrations were initially low following fruit set and rose steadily to over 40% by harvest (*P* < 0.001). Mean sugar concentrations increased more than three fold over the same period (*P* < 0.001). Total NSC concentrations followed the same trajectory, reaching ~78% by harvest (*P* < 0.001). In contrast to a lack of significant treatment × date effects on sucrose:hexose ratio in fruit during both seasons (*P* > 0.05; Figure 3), fruit from previously defoliated trees exhibited a significant increase across dates in 2024–25 relative to controls (*P* = 0.026).

### Non-structural carbohydrate reserve responses to acute carbon limitation (2023–24)

#### Starch dynamics

**Figure 4:**
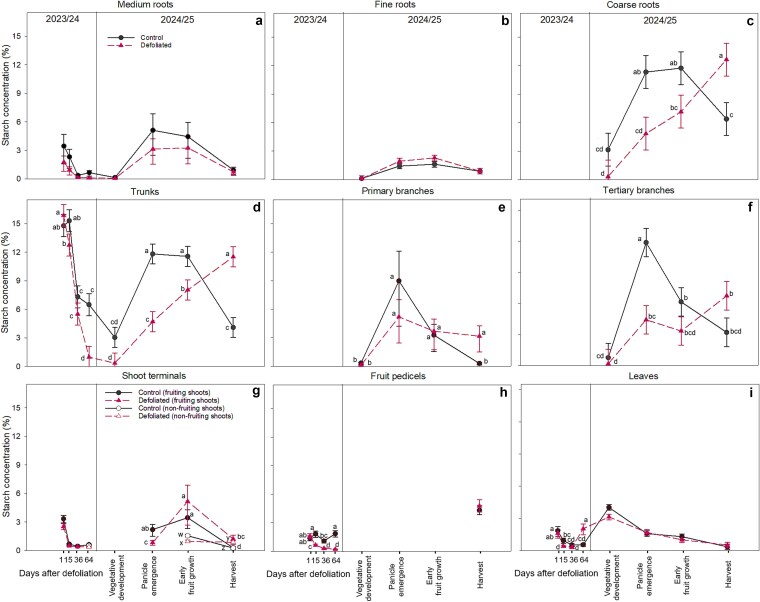
Seasonal dynamics of starch concentrations (%) in different organs of control and defoliated trees during the 2023–24 and 2024–25 seasons. Panels show (a) medium roots, (b) fine roots, (c) coarse roots, (d) trunks, (e) primary branches, (f) tertiary branches, (g) shoot terminals (fruiting and non-fruiting), (h) fruit pedicels and (i) leaves. Points represent treatment means, and error bars indicate ±1 standard error of the mean. Different lowercase letters within each season indicate statistically significant treatment × sampling time interactions (*P* < 0.05).

Under acute source limitation, starch dynamics diverged among organs (Figure 4). Of the tissues assessed, trunk wood exhibited the clearest treatment response, with a significant treatment × date interaction (*P* = 0.013; Figure 4d). Starch concentrations declined more sharply in defoliated trees than in controls, resulting in approximately seven fold lower concentrations at harvest. Total NSC concentrations in trunks closely paralleled starch, with a similarly significant treatment × date interaction (*P* = 0.005; see Figure S1 available as Supplementary Data at *Tree Physiology* Online). In medium roots, starch concentrations declined significantly over time in both treatments up to 36 days after defoliation and then stabilized (*P* < 0.001), with a modest overall treatment effect (*P* = 0.044) in line with higher concentrations maintained in control trees (Figure 4a). No significant treatment × date interaction was detected (*P* > 0.05). Mean total NSC concentrations in medium roots followed the same temporal pattern (*P* < 0.001; see Figure S1 available as Supplementary Data at *Tree Physiology* Online).

In tissues proximal to fruit, starch responses varied less distinctly but were still treatment-sensitive. In fruiting shoots, starch concentrations were significantly influenced by collection date (*P* < 0.001) and treatment (*P* = 0.012) (Figure 4g). Starch declined sharply within 15 days after defoliation and stabilized at lower levels, with controls maintaining modestly higher concentrations. In non-fruiting shoots, starch at harvest did not differ significantly between treatments (*P* = 0.260; Figure 4g). In fruit pedicels, starch concentrations showed a strong treatment × date interaction (*P* < 0.001; Figure 4h). Starch declined rapidly in defoliated trees by 15 days after defoliation and remained lower, such that by harvest controls maintained approximately nine fold higher concentrations. Leaf starch concentrations were also influenced by a significant treatment × date interaction (*P* < 0.001; Figure 4i). Starch declined in both treatments by 15 days after defoliation, with lower concentrations in defoliated trees at that stage. Thereafter, starch remained stable in controls but increased in defoliated trees, such that by harvest defoliated trees exhibited approximately four fold higher leaf starch concentrations.

#### Soluble sugar dynamics

**Figure 5:**
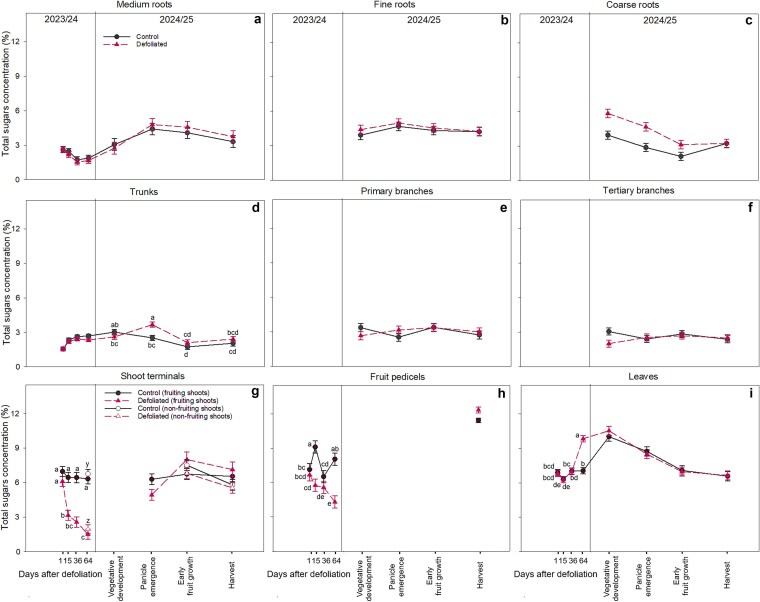
Seasonal dynamics of total soluble sugar concentrations (%) in different organs of control and defoliated trees during the 2023–24 and 2024–25 seasons. Panels show (a) medium roots, (b) fine roots, (c) coarse roots, (d) trunks, (e) primary branches, (f) tertiary branches, (g) shoot terminals (fruiting and non-fruiting), (h) fruit pedicels and (i) leaves. Points represent treatment means, and error bars indicate ±1 standard error of the mean. Different lowercase letters within each season indicate statistically significant treatment × sampling time interactions (*P* < 0.05).

Treatment effects on soluble sugars were most evident in sugar-dominant tissues, namely fruiting shoot terminals, fruit pedicels and leaves (Figure 5). Mean sugar concentrations showed significant treatment × date interactions in fruiting shoots and leaves (both *P* < 0.001) and in pedicels (*P* = 0.002). In fruiting shoots, sugars declined sharply in defoliated trees, whereas controls maintained higher concentrations throughout, reaching more than four fold greater values at harvest (Figure 5g). Non-fruiting shoots at harvest similarly exhibited higher sugar concentrations in controls (*P* < 0.001) (Figure 5g). Mean total NSC concentrations in shoots mirrored these sugar dynamics, with a significant treatment × date interaction (*P* = 0.021; see Figure S1 available as Supplementary Data at *Tree Physiology* Online). In pedicels, sugar concentrations declined over time after defoliation and were nearly double in control trees compared with defoliated trees at harvest (Figure 5h). Total NSC concentrations mirrored this pattern, with a significant treatment × date interaction (*P* < 0.001; see Figure S1 available as Supplementary Data at *Tree Physiology* Online). In leaves, sugars initially increased in both treatments and continued to rise in defoliated trees, such that concentrations exceeded controls by harvest (Figure 5i). Mean total NSC concentrations mirrored these sugar trends and were significantly influenced by the treatment × date interaction (*P* < 0.001; see Figure S1 available as Supplementary Data at *Tree Physiology* Online). In contrast, perennial storage tissues such as medium roots (Figure 5a) and trunks (Figure 5d) showed no significant treatment effects on mean total sugar concentrations (*P* > 0.05).

#### Sucrose:hexose ratios

Sucrose:hexose ratios showed tissue-specific responses, with significant treatment × date interactions in fruiting shoots (*P* = 0.002) (Figure 3). Defoliated trees exhibited lower ratios shortly after defoliation but transiently exceeded control values by 36 days. In non-fruiting shoots, ratios were lower in defoliated trees at harvest (*P* = 0.009). In pedicels, no significant interaction was detected, but controls maintained higher ratios across dates (*P* = 0.002). In leaves, sucrose:hexose ratios showed a significant treatment × date interaction (*P* = 0.003), remaining stable in controls but increasing progressively in defoliated trees. In medium roots, a significant treatment × date interaction was detected (*P* = 0.003), with ratios increasing in controls but declining in defoliated trees. In trunks, no interaction was detected, but controls maintained higher ratios across sampling dates (*P* = 0.005).

### Non-structural carbohydrate reserve dynamics following prior defoliation (2024–25)

#### Starch dynamics

Root starch dynamics differed among root types and growth stages during the second season (Figure 4). In medium roots, starch concentrations varied significantly with sampling date (*P* < 0.001), peaking around panicle emergence and early fruit growth before declining by harvest (Figure 4a). Total NSC concentrations followed the same temporal pattern (see Figure S1 available as Supplementary Data at *Tree Physiology* Online). In fine roots, mean starch concentrations peaked around panicle emergence and early fruit growth before declining by harvest (Figure 4b). Previously defoliated trees tended to have slightly higher starch (*P* = 0.074), while total NSC concentrations were significantly higher in previously defoliated trees across sampling dates (*P* = 0.014; see Figure S1 available as Supplementary Data at *Tree Physiology* Online). There was a significant treatment × date interaction for mean starch in coarse roots (*P* = 0.003; Figure 4c). Total NSC concentrations showed a similar interaction (*P* = 0.008; see Figure S1 available as Supplementary Data at *Tree Physiology* Online). Starch and total NSC concentrations in coarse roots were initially low and similar between groups. From panicle emergence, controls showed higher concentrations. By early fruit growth the difference narrowed, but by harvest, previously defoliated trees contained nearly twice as much starch as controls.

Woody trunk and branch tissues showed broadly similar treatment-dependent starch trajectories. A significant treatment × date interaction was detected for mean trunk starch (Figure 4d) and total NSC concentrations (both *P* < 0.001; see Figure S1 available as Supplementary Data at *Tree Physiology* Online). Starch concentrations increased from early vegetative growth to panicle emergence. In controls, starch plateaued by early fruit growth and declined by harvest, whereas previously defoliated trees continued accumulating starch, reaching levels three fold higher than controls at harvest. Total NSC concentrations closely mirrored starch trends. Mean starch concentrations in primary branches showed a significant interaction between treatment and collection date (*P* < 0.001; Figure 4e). Starch levels were initially low. Controls peaked at panicle emergence then declined, whereas previously defoliated trees remained relatively stable after initial accumulation. By harvest, previously defoliated trees exceeded controls more than ten fold. Tertiary branches showed similar starch dynamics, with a significant treatment × date interaction (*P* = 0.002; Figure 4f). Mean total NSC concentrations in primary and tertiary branches were also influenced by treatment × date interactions (both *P* = 0.006; see Figure S1 available as Supplementary Data at *Tree Physiology* Online), mirroring the starch responses.

Starch concentrations in fruiting and non-fruiting shoots varied significantly with treatment × date interactions (*P* = 0.009 and *P* = 0.004, respectively; Figure 4g). By harvest, starch concentrations in control trees were lower than in previously defoliated trees for both shoot types. In pedicels, neither starch (*P* = 0.558; Figure 4h) nor total NSC concentrations (*P* = 0.136; see Figure S1 available as Supplementary Data at *Tree Physiology* Online) differed significantly between treatments at harvest. Leaf starch concentrations declined progressively across developmental stages (*P* < 0.001), with no significant treatment effect (Figure 4i).

#### Soluble sugar dynamics

Seasonal effects on mean total soluble sugars were evident across several tissues (Figure 5). In medium roots, sugar concentrations were significantly affected by sampling date (*P* = 0.002), increasing initially and then declining during reproductive development (Figure 5a). In coarse roots, sugar concentrations declined until early fruit growth before stabilizing by harvest (*P* < 0.001; Figure 5c). In coarse roots, sugar levels were higher in previously defoliated trees across sampling dates (*P* = 0.002). In fine roots, previously defoliated trees tended to show higher sugar concentrations (*P* = 0.086; Figure 5b). In trunks, a significant treatment × date interaction was detected (*P* = 0.027; Figure 5d), with the clearest difference at panicle emergence, where previously defoliated trees exhibited higher sugar concentrations than controls. At other dates, differences were minor. Mean total sugar concentrations in primary and tertiary branches were not significantly influenced by sampling date or treatment (*P* > 0.05; Figure 5e and f).

In fruiting and non-fruiting shoots, sugar concentrations were significantly affected by sampling date (*P* = 0.021 and *P* = 0.007, respectively; Figure 5g). In fruiting shoots, sugars increased from panicle emergence to early fruit growth before plateauing. Total NSC concentrations in fruiting shoots showed a significant treatment × date interaction (*P* = 0.018; see Figure S1 available as Supplementary Data at *Tree Physiology* Online), mirroring sugars. In non-fruiting shoots, sugars declined during fruit growth in both treatments. In pedicels, mean total sugar concentrations were marginally higher in previously defoliated trees at harvest (*P* = 0.054; Figure 5h). In leaves, sampling date significantly affected total sugar and total NSC concentrations (both *P* < 0.001; Figure 5i; Figure S1 available as Supplementary Data at *Tree Physiology* Online), which declined progressively toward harvest without significant treatment effects.

#### Sucrose:hexose ratios

A significant treatment × date interaction was detected for sucrose:hexose ratios in trunks (*P* < 0.001; Figure 3). Control trees exhibited a transient increase in the ratio at panicle emergence, whereas previously defoliated trees showed higher ratios by harvest. In primary branches, sucrose:hexose ratios were influenced by a significant treatment × date interaction (*P* = 0.006), with higher ratios in previously defoliated trees at harvest. No consistent treatment effects on sucrose:hexose ratios were detected in most other tissues.

### Whole-tree coordination of seasonal NSC dynamics

**Figure 6:**
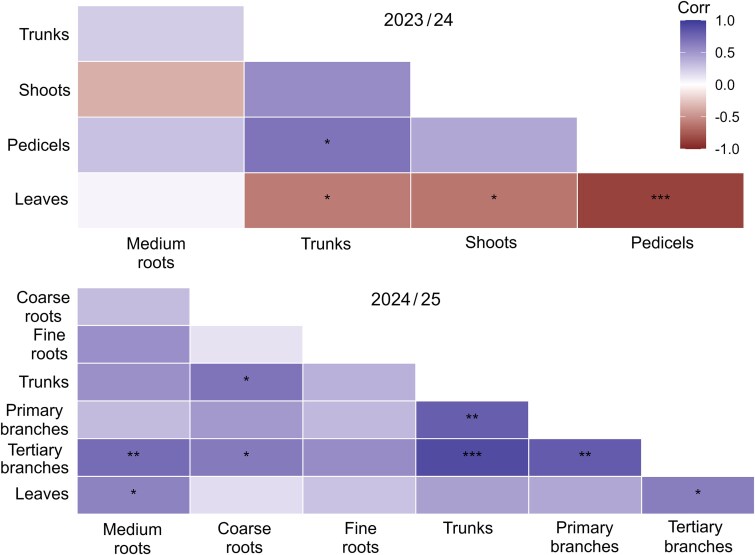
Spearman rank correlations among seasonal changes in total non-structural carbohydrate concentration (ΔNSC) across organs. ΔNSC was calculated as the difference between the final and first sampling dates within each season. Only organs sampled across the entirety of each season were included (2023–24: medium roots, trunk wood, fruiting shoot terminals, fruit pedicels and leaves; 2024–25: medium, fine and coarse roots, trunk wood, primary and tertiary branches and leaves). Correlation strength and direction are represented by the heatmap scale, with positive values indicating positive correlations and negative values indicating negative correlations. Asterisks denote significance levels (^*^P < 0.05, ^**^P < 0.01, ^***^P < 0.001).

In the 2023–24 season, ΔNSC in trunk wood was significantly positively correlated with ΔNSC in fruit pedicels and significantly negatively correlated with ΔNSC in leaves (both *P* = 0.015; Figure 6). The positive association between trunk wood and fruiting shoot terminals approached significance (*P* = 0.063). ΔNSC in fruiting shoot terminals was significantly negatively correlated with ΔNSC in leaves (*P* = 0.031), and ΔNSC in pedicels was strongly negatively correlated with ΔNSC in leaves (*P* < 0.001).

In the 2024–25 season, ΔNSC in medium roots was positively correlated with ΔNSC in leaves (*P* = 0.039) and tertiary branches (*P* = 0.001), and showed near-significant positive correlations with fine roots (*P* = 0.071) and trunk wood (*P* = 0.075). ΔNSC in coarse roots was significantly positively correlated with ΔNSC in trunk wood (*P* = 0.015) and tertiary branches (*P* = 0.022). Fine-root ΔNSC also showed near-significant positive correlations with medium roots (*P* = 0.071) and tertiary branches (*P* = 0.063). Trunk ΔNSC was significantly positively correlated with both primary (*P* = 0.002) and tertiary branches (*P* < 0.001). Primary-branch ΔNSC was positively correlated with tertiary branches (*P* = 0.001), while tertiary-branch ΔNSC was also positively correlated with leaves (*P* = 0.028). Full Spearman correlation coefficients and associated *P*-values for each season are provided (see Table S2 available as Supplementary Data at *Tree Physiology* Online).

## Discussion

### Reproductive responses to acute carbon limitation and subsequent recovery

Fruit growth was consistently reduced in trees subjected to defoliation, confirming the reliance of mango fruit development on ongoing photosynthetic supply (Léchaudel et al. 2005a). The quadratic regression reflected a sustained decline in fruit sink activity under acute source limitation (Sonnewald and Fernie 2018). The stabilization of fruit growth rate during the final three weeks before harvest coincided with a major rainfall event (see Figure S2 available as Supplementary Data at *Tree Physiology* Online). Although complicated by interactions between leaf-to-fruit ratio and tree water status, increased soil water availability can enhance mango fruit expansion, largely by improving fruit turgor (Léchaudel et al. 2005*b*, Spreer et al. 2009). Thus, the rainfall event may have contributed to the observed stabilization of fruit growth rate in both treatments. Nevertheless, reserve mobilization partially buffered fruit growth under severe canopy loss, yet proved insufficient to sustain reproductive sink strength in the absence of continued carbon assimilation (Rossouw et al. 2018). Previous studies have similarly shown that mango fruit growth is sensitive to source–sink imbalances, with lower leaf-to-fruit ratios leading to reduced fruit size (Chacko et al. 1982, Léchaudel and Joas 2006). The present findings extend this by quantifying such growth suppression under near-complete canopy loss.

Carbon source limitation imposed by late-season defoliation had pronounced effects on fruit yield and NSC status. Reductions in fruit number, size, dry matter and starch accumulation meant that total NSC deposition in fruit from defoliated trees was only about one-third that of controls. Fruit NSC accumulation was thus strongly curtailed, with canopy assimilation proving critical to sustain deposition (Chacko et al. 1982, Léchaudel et al. 2007). This reduction was underpinned by divergent fruit starch accumulation between treatments, indicating that control trees maintained assimilate influx and starch synthesis during late fruit development, whereas defoliated trees showed a transient drop followed by stagnation. The elevated sugar concentrations in defoliated-tree fruit are consistent with carbon stress responses, where reduced assimilation favors soluble sugar retention over starch storage (Lemoine et al. 2013, Ma et al. 2025). This likely reflects impaired starch biosynthesis and preferential allocation of sugars to sustain residual sink activity (Ren et al. 2023).

In the 2024–25 season, fruit NSC concentrations recovered, indicating restored canopy function and assimilate supply. However, yield remained significantly lower, due to reduced fruit number, with only three of six previously defoliated trees producing fruit. This suggests that prior-season carbon stress had lingering effects on floral initiation, inflorescence development and/or early fruit retention (Smith and Samach 2013, Cho et al. 2018, Rossouw et al. 2024*a*). At the fruit level, however, starch and sugar concentrations increased steadily in both treatments, yet fruit on previously defoliated trees exhibited higher starch concentrations and dry matter content in the second season. These trends likely reflect reduced competition for assimilates in trees carrying lower fruit loads (Ngao et al. 2020), consistent with evidence in mango and other fruit trees that reduced reproductive demand enhances per-fruit NSC allocation (Léchaudel and Joas 2006).

The sucrose:hexose ratio provides additional insight into fruit sink metabolism (Lee et al. 2021). In developing fruit, a low sucrose:hexose ratio is typically associated with strong sink activity, reflecting high invertase-mediated cleavage of imported sucrose to sustain cell expansion (Ren et al. 2023, Rossouw et al. 2024*b*). In the present study, the sucrose:hexose ratio tended to be higher in fruit from previously defoliated trees during the recovery season. A relative increase in sucrose proportion may indicate reduced sucrose cleavage and lower metabolic demand. Consistent with this interpretation, the sucrose:hexose ratio generally increases as fruit growth slows and development transitions from cell expansion to maturation (Lee et al. 2021). The elevated ratio observed here is consistent with reduced crop load and relatively larger fruit and higher dry matter content, suggesting that fruit on previously defoliated trees may have progressed more rapidly toward physiological maturity.

These findings indicate that while NSC reserves can temporarily buffer reproductive sink demand, sustained canopy assimilation is required to maintain both fruit development and long-term reproductive capacity. When assimilation is severely curtailed, carbon allocation shifts away from reproductive developmental support, leading to immediate yield suppression and longer-term yield instability. Under well-functioning canopy conditions, reserves can support high crop loads by smoothing seasonal supply–demand imbalances; however, when assimilation is suppressed, the negative repercussions for yield and fruit quality can compound across successive seasons.

### Reserve mobilization during acute carbon limitation

Acute canopy loss triggered coordinated adjustments in NSC reserves across perennial woody storage organs, roots and sugar-dominated tissues, including shoots, pedicels and leaves. Mango trees comprise structurally and functionally distinct storage compartments, including trunk wood, coarse roots, branches and smaller root classes, each seemingly contributing differently to reserve buffering and remobilization (Davie et al. 1999, Normand et al. 2009). During the defoliation season, reserve depletion proved most pronounced in long-term storage tissues such as the trunk, whereas in the subsequent recovery season, reserve rebuilding occurred synchronously across perennial organs, including roots, trunk and branches, under reduced reproductive demand.

During the defoliation season, starch reserves in woody storage organs were heavily drawn upon to buffer reproductive demand when canopy assimilation was curtailed. Starch in trunks of defoliated trees declined sharply, culminating in concentrations substantially lower than controls by harvest. This pronounced depletion highlights reliance on trunk starch to buffer reproductive demand under acute carbon limitation (Loescher et al. 1990, Davie and Stassen 1997). Although NSC levels in coarse roots and branches were not quantified during the defoliation season, the magnitude of trunk depletion suggests that central storage compartments with high NSC concentrations were heavily mobilized under severe source limitation. Medium-sized roots also exhibited seasonal declines in starch, which, as in the trunk, constituted the dominant component of the NSC pool; however, treatment effects were not clearly evident. Across the season, medium-root starch dynamics largely reflected phenological evolution rather than defoliation per se, indicating a more transient buffering role compared with trunk reserves (Hartmann and Trumbore 2016, Klein et al. 2016). The absence of strong treatment effects suggests that medium roots were not preferentially depleted under canopy loss. Sucrose:hexose ratios further resolved these seasonal adjustments in woody and root tissues. In both trunks and medium roots, lower ratios in defoliated trees across sampling dates suggest enhanced sucrose cleavage under acute carbon limitation, potentially supporting maintenance respiration and metabolic demand (Khanna et al. 2022). Given the modest changes in absolute sugar concentrations in medium roots, these ratio shifts likely reflect short-term metabolic modulation rather than a substantive reconfiguration of long-term reserve allocation.

In contrast to perennial woody organs, sugar concentrations in sugar-dominated tissues responded rapidly and distinctly to canopy loss. In fruiting shoots, defoliation triggered marked depletion of sugars, with concentrations falling to less than one-quarter of control values by harvest. Although starch declined similarly in controls, the pronounced depletion of sugars in defoliated trees likely reflects sustained export of readily mobilizable carbon to support fruit sinks under reduced photosynthetic supply (Ren et al. 2023). Likewise, by harvest, non-fruiting shoots in defoliated trees exhibited markedly lower sugar concentrations, suggesting that non-fruiting terminals contributed to systemic mobilization in support of fruiting tissues (Capelli et al. 2021). The transient reduction in sucrose:hexose ratio in fruiting terminals shortly after defoliation was also consistent with accelerated sucrose utilization and export under sudden carbon limitation, rather than a sustained shift in sugar metabolism (Lemoine et al. 2013, Sonnewald and Fernie 2018).

In fruit pedicels, sharp declines in both starch and sugars in trees subjected to defoliation highlight the vulnerability of these tissues under acute source limitation. Fruit pedicels appear to function as both assimilate conduits and short-term buffering sites, linking source tissues with developing fruit (Ma et al. 2019). Lower sucrose:hexose ratios in pedicels of defoliated trees suggest enhanced sucrose export under acute source limitation. Because sucrose is the principal form of carbon transported in mango phloem (Rossouw et al. 2024*b*), a reduction in sucrose proportion is consistent with intensified flux toward fruit sinks. Together with starch depletion, this pattern suggests that pedicels not only mobilized local reserves but also increased sucrose flux toward developing fruit under carbon stress. Consistently lower pedicel NSC status under defoliation may also have contributed to the reduced fruit numbers observed at harvest. Insufficient assimilate supply at the pedicel and abscission zone is known to promote premature fruit abscission (Jones et al. 2026), and the observed NSC depletion is consistent with this mechanism under carbon limitation.

The few remaining leaves on defoliated trees accumulated progressively higher starch and sugar concentrations by harvest, ultimately exceeding control values. This pattern indicates a compensatory response to canopy loss, with enhanced assimilate accumulation in the remaining foliage under severe source limitation. Such accumulation has been described in other tree species as part of an acclimation strategy, enabling leaves to partly sustain sink demand when overall functional leaf area is impaired (El Omari 2022, Luo et al. 2024). The accompanying increase in sucrose:hexose ratio in leaves of defoliated trees further supports this compensatory interpretation. Because sucrose is the principal export form of carbon in mango leaves, a higher sucrose proportion relative to hexoses is consistent with enhanced source activity and export potential within the reduced canopy (Lemoine et al. 2013, Rossouw et al. 2017*a*).

Whole-tree coordination analyses were consistent with this integrated mobilization pattern. Although these correlations do not establish causal relationships or direct carbon transfer between tissues, they provide evidence of coordinated variation in seasonal NSC dynamics among organs. During the defoliation season, ΔNSC correlations showed that changes in trunk wood were positively associated with changes in fruit pedicels, consistent with coordinated reserve remobilization between central woody tissues and reproductive structures when canopy assimilation was severely curtailed (Rossouw et al. 2017*b*, Huang et al. 2023). In contrast, negative associations between leaves and both pedicels and fruiting shoots reflect compensatory sugar accumulation in residual foliage concurrent with NSC depletion in reproductive-supporting tissues. These opposing relationships are consistent with coordinated but functionally differentiated responses across the tree, linking trunk depletion, limited medium-root treatment sensitivity, shoot and pedicel export, and leaf-level compensation within a unified whole-tree allocation response (Wang et al. 2021).

### Coordinated reserve rebuilding during canopy recovery

In contrast to the pronounced reserve mobilization observed under acute canopy loss, the second season was characterized by coordinated reserve rebuilding across organs. Canopy function appeared restored, yet reproductive demand remained reduced in previously defoliated trees, creating conditions favorable for reserve reconstitution (Rossouw et al. 2024*b*).

Trunk and coarse root reserves exhibited the clearest evidence of systemic rebuilding. In trunks, previously defoliated trees showed continued accumulation of starch and total NSCs through to harvest, ultimately finishing two- to three fold higher than controls. Unlike the sharp depletion observed during the defoliation season, trunk reserves in previously defoliated trees were not mobilized late in fruit development. Instead, their progressive accumulation suggests a reallocation strategy favoring storage over reproduction, facilitated by lower fruit loads (Wiley et al. 2017). In contrast, control trees replenished trunk starch early but subsequently mobilized reserves during fruit growth, consistent with their higher reproductive demand (Rossouw et al. 2017*a*). These contrasting trajectories across seasons highlight the trunk as a central integrator of the trade-off between carbon storage and reproduction. Treatment-specific shifts in sucrose:hexose ratios in trunks mirrored starch rebuilding trajectories. In controls, the transient elevation of the ratio by panicle emergence coincided with early starch replenishment, whereas in previously defoliated trees, higher ratios later in the season aligned with their prolonged starch accumulation. Increases in the relative sucrose proportion during phases of starch accumulation are consistent with enhanced sucrose import and conversion to starch for storage (Loescher et al. 1990, Sala et al. 2012). These temporally aligned dynamics suggest a shift in trunk function from reserve buffering under carbon limitation to reserve consolidation during recovery.

Primary and tertiary branches showed parallel reserve replenishment patterns. In controls, starch rose early and declined during fruit development, consistent with mobilization to sustain reproductive sinks. Previously defoliated trees, however, maintained or gradually increased starch through to harvest, finishing with higher reserve levels. The lower fruit load in previously defoliated trees appears to have favored reserve replenishment rather than mobilization (Rossouw et al. 2024*b*, Poupard et al. 2025). These dynamics mirror those in trunks, albeit less pronounced and suggest that branches function as intermediate storage pools capable of both seasonal buffering and longer-term reserve recovery (Loescher et al. 1990). As in trunks, elevated sucrose:hexose ratios in primary branches of previously defoliated trees by harvest are consistent with reserve consolidation under reduced sink demand. Collectively, these results suggest that, following severe carbon source limitation, mango trees re-prioritize NSC allocation toward stabilizing and rebuilding reserves in woody structural tissues (Bruce et al. 2007, Sala et al. 2012).

Root responses revealed a functional gradient in storage capacity. Coarse roots showed the strongest treatment responsiveness. Control trees replenished starch early but depleted it during fruiting, whereas previously defoliated trees accumulated starch more gradually and finished with greater reserves. This delayed yet stronger accumulation is consistent with compensatory reserve rebuilding under reduced reproductive investment (Le Roncé et al. 2020) and supports the role of coarse roots as major long-term storage sites in mango (Stassen and Janse van Vuuren 1997). Previously defoliated trees also tended to maintain higher sugar concentrations early in the season, which declined as starch reserves accumulated. This inverse relationship between soluble sugars and starch is consistent with progressive conversion of imported sugars into storage forms during reserve rebuilding (Sala et al. 2012, El Omari 2022). Medium roots, by contrast, exhibited phenology-driven fluctuations similar to those observed in the previous season. Starch levels replenished prior to panicle emergence and declined during fruiting, but treatment effects were modest. Across all root classes, the absence of treatment effects on sucrose:hexose ratios indicates that recovery was expressed primarily through starch reserve dynamics rather than sustained alterations in sugar composition.

Fine roots, however, displayed subtle but informative differences. Previously defoliated trees maintained somewhat higher NSC concentrations during recovery. Given that fine roots are metabolically active, low-storage organs with high turnover and construction costs (McCormack et al. 2014), this outcome likely reflects altered whole-tree carbon balance rather than targeted reallocation (Zhao and Gong 2021). The dominance of soluble sugars in fine roots points to a role in short-term NSC fluxes and osmotic regulation rather than long-term storage (Ciereszko 2018). Transiently elevated NSC concentrations in fine roots of previously defoliated trees may therefore arise through more than one mechanism. One possibility is raised carbon availability under reduced reproductive sink demand. Alternatively, higher NSC concentrations may reflect changes in utilization rather than increased allocation. Stored carbon in fine roots can contribute substantially to respiratory demand without being heavily invested in new root production (Lynch et al. 2013), and carbon consumption reflects a dynamic balance between anabolic and catabolic processes that varies with root anatomy, physiology and environmental conditions (Valverde-Barrantes 2022). From this perspective, the observed differences in fine-root NSC concentrations are best interpreted as emergent properties of whole-tree carbon balance during recovery rather than evidence of a targeted shift in allocation priority.

Consistent with these organ-level trajectories, whole-tree ΔNSC correlations in the second season shifted toward positive associations among perennial woody tissues. Trunk wood, branches and coarse roots all exhibited positive relationships, consistent with synchronous reserve replenishment within the perennial framework and supporting the interpretation that these tissues function as an integrated reserve network rather than isolated storage pools (Zahnd et al. 2024). This system-level coordination is consistent with the view that, once carbon supply is restored and crop load reduced, carbon allocation is preferentially directed toward rebuilding the structural reserve base of the tree.

In contrast to woody storage organs, fruit-adjacent tissues exhibited largely restored NSC dynamics during the recovery season, with fewer persistent differences between treatments. Among these, fruiting and non-fruiting shoot terminals maintained sugar-dominant profiles consistent with their role as active growth sinks, where sugar metabolism supports respiration, biosynthesis and sustained apical meristem activity (Su et al. 2022). Starch fluctuations appeared to contribute modestly to buffering source–sink imbalances (Sala et al. 2012). In previously defoliated trees, starch accumulation in shoots increased by harvest, reflecting lower fruit load and a shift toward reserve consolidation. Control trees, carrying heavier crops, depleted shoot starch during fruit development. These patterns mirror those observed in branches and coarse roots, and highlight that shoots act as short-term NSC supply hubs for nearby developing fruit (Capelli et al. 2021, Rossouw et al. 2024*a*).

Pedicel NSC concentrations no longer differed significantly between treatments, indicating that depletion observed in the defoliation season was transient rather than persistent. Restoration of pedicel NSC status suggests that source–sink coordination within new panicles was re-established once canopy function recovered (Ma et al. 2019). Leaf NSC concentrations followed similar temporal trajectories in both treatments. Despite differences in crop load, leaf NSC status appeared buffered from systemic imbalance once canopy supply was restored. This contrasts sharply with the compensatory accumulation observed under acute defoliation and indicates that foliar adjustments were reversible and stress-specific (Iglesias et al. 2002, Iqbal et al. 2012). Together, the recovery-season patterns indicate a functional separation among organ groups: trunks, branches and coarse roots accumulated reserves; smaller root classes showed more transient or metabolically mediated responses; and fruit-adjacent tissues and leaves largely regained NSC dynamics observed before carbon limitation.

### Whole-tree allocation dynamics across successive seasons

These results support a conceptual pathway in which late-season defoliation drives extensive depletion of NSC reserves during the first season, constraining reproductive investment, followed by a second-season shift toward synchronous reserve rebuilding across woody organs under reduced crop load. By explicitly quantifying associations among organs, the whole-tree ΔNSC analysis extends organ-specific responses into a more integrative framework of carbon allocation, showing patterns consistent with the view that transient carbon limitation can influence reproductive outcomes across successive seasons.

The integrated whole-tree reserve allocation patterns observed here may have broader relevance beyond mango. Tree species differ in phenology, crop load and storage anatomy, including variation in xylem parenchyma abundance and plastid-rich tissues that influence NSC storage capacity and remobilization (Zhang et al. 2022). Nevertheless, many perennial crops, including apple, citrus (*Citrus* spp.) and grapevine, similarly rely on trunks, roots and perennial branches as long-term NSC reservoirs that buffer seasonal imbalances between carbon supply and reproductive demand (Rossouw et al. 2017*a*, Agustí et al. 2022, Plavcová et al. 2024). Orchard management strategies that protect canopy function and support reserve replenishment during critical phenological windows such as post-harvest may enhance resilience across seasons. While the magnitude and timing of these responses will vary among species, the underlying trade-off between reproduction and reserve maintenance remains a central determinant of perennial crop performance, particularly in abiotic stress-prone production systems.

## Conclusions

This study demonstrates that mango fruit development depends strongly on canopy photosynthesis, with NSC reserves only partly compensating when photosynthetic capacity is curtailed. Severe late-season defoliation suppressed fruit growth, yield and starch deposition, and reproductive penalties carried over into the following season, reducing fruit number and yield despite apparent recovery of a functional canopy. Organ-specific reserve dynamics point to a functional hierarchy based on turnover and recovery behavior rather than absolute pool size: coarse roots and trunks exhibited characteristics consistent with long-term starch storage, primary and tertiary branches provided intermediate buffering, and medium roots served as more transient storage organs, responding mainly to phenological demand. Fine roots, shoots, pedicels and leaves, as sugar-dominant tissues, functioned as short-term nodes of supply and adjustment. Tissue-specific shifts in sucrose:hexose balance further clarified these organ-level responses to carbon limitation. During acute defoliation stress, roots and trunks tended to have reduced sucrose proportions, consistent with enhanced sucrose utilization in woody, starch-dominant tissues, while fruiting terminals and pedicels showed lower sucrose:hexose ratios aligned with rapid export of readily available transport sugars to sustain fruit sink demand. In the subsequent season, previously depleted storage organs, particularly coarse roots and trunks, exhibited strong reserve replenishment, highlighting a shift in carbon allocation priorities from reproduction toward reserve restoration. Correlation analyses of seasonal NSC changes further support the interpretation that recovery involved coordinated variation across perennial woody organs, rather than isolated, organ-specific responses. These findings emphasize the centrality of canopy assimilate supply in sustaining reproductive output and the limited capacity of reserves to buffer prolonged carbon stress. Importantly, a single season of carbon limitation can impose multi-seasonal impacts on yield and carbon allocation strategies. These insights provide a physiological basis for canopy and crop-load management to stabilize yields and mitigate alternate bearing.

## Supplementary Material

Supplementary_data_tpag069

## Data Availability

Data are available at the discretion of the corresponding author upon reasonable request.
