# Supplementary material for: Carbohydrate reserve partitioning and reproductive decline following defoliation-induced carbon source limitation in mango (Mangifera indica)
Source: Tree Physiol. 2026 May 22;46(6):tpag069. doi: 10.1093/treephys/tpag069 (PMC13310119; doi:10.1093/treephys/tpag069)
Supplement: Supplementary_data_tpag069 [file supplementary_data_tpag069.pdf]

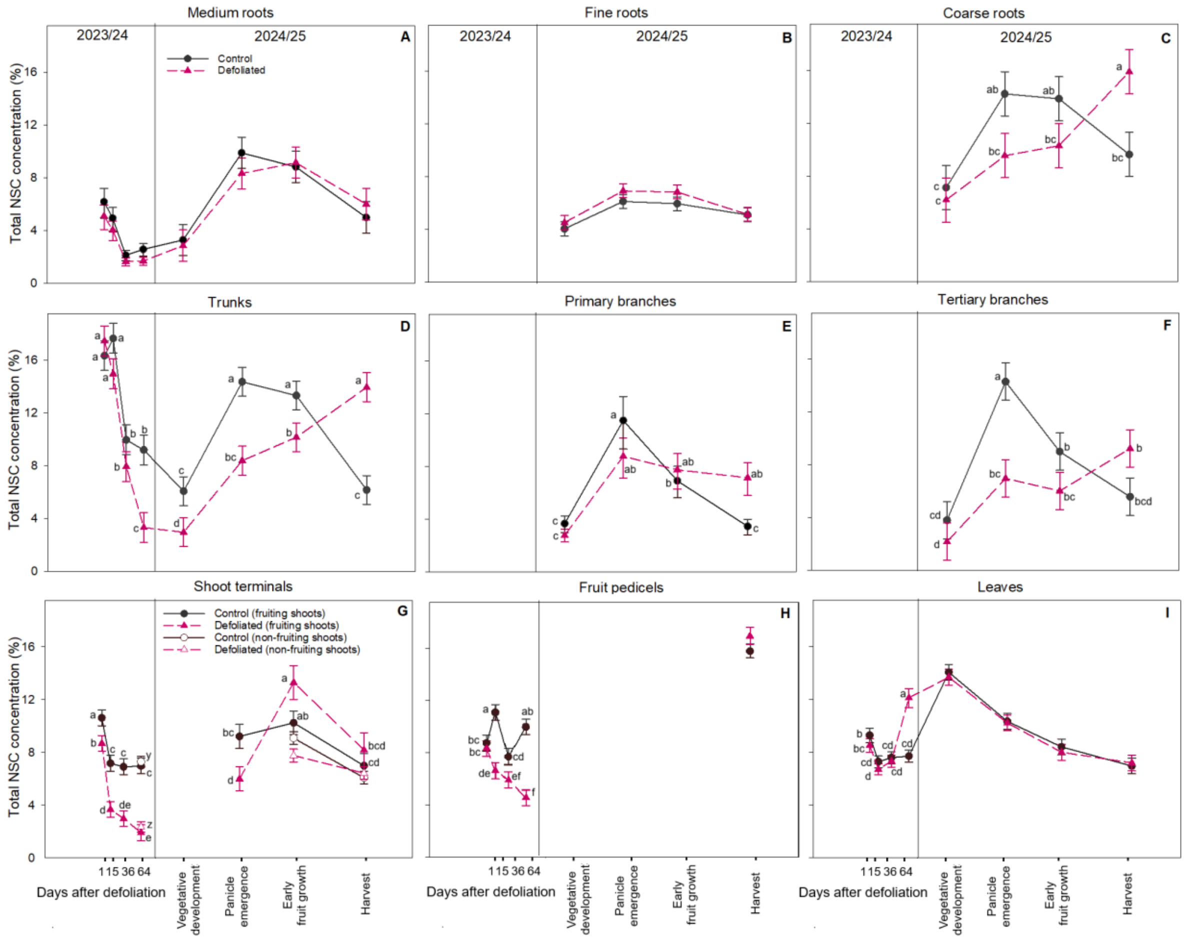

Supplementary figure S1: Seasonal dynamics of total non-structural carbohydrate concentrations (%) in different organs of control and defoliated trees during the 2023–2024 and 2024–2025 seasons. Panels show (A) medium roots, (B) fine roots, (C) coarse roots, (D) trunks, (E) primary branches, (F) tertiary branches, (G) shoot terminals (fruiting and non-fruiting), (H) fruit pedicels, and (I) leaves. Points represent treatment means, and error bars indicate ± 1 standard error of the mean. Different lowercase letters within each season indicate statistically significant treatment × sampling date interactions ($P < 0.05$).

Supplementary table S1: Summary statistics for sucrose, glucose, and fructose concentrations (%) in each organ of control and defoliated trees across sampling dates in the 2023–2024 and 2024–2025 seasons. For each organ × treatment × sampling date combination, the table reports mean, standard deviation (SD), standard error (SE), and sample size (n), calculated from available biological replicates (maximum n = 6).

Season	Sampling time	Treatment	Organ	Sugar	n	Mean	Standard Deviation	Standard Error
2023-24	1 day after defoliation	Control	Fruit	Fructose	6	12.88	1.668	0.681
2023-24	1 day after defoliation	Defoliated	Fruit	Fructose	6	12.53	1.341	0.547
2023-24	15 days after defoliation	Control	Fruit	Fructose	6	13.46	1.749	0.714
2023-24	15 days after defoliation	Defoliated	Fruit	Fructose	6	15.19	0.691	0.282
2023-24	36 days after defoliation	Control	Fruit	Fructose	6	13.45	0.779	0.318
2023-24	36 days after defoliation	Defoliated	Fruit	Fructose	6	15.40	1.727	0.705
2023-24	64 days after defoliation	Control	Fruit	Fructose	6	13.92	1.967	0.803
2023-24	64 days after defoliation	Defoliated	Fruit	Fructose	6	17.95	2.202	0.899
2024-25	Early fruit growth	Control	Fruit	Fructose	6	3.60	0.789	0.322
2024-25	Early fruit growth	Defoliated	Fruit	Fructose	3	2.70	1.078	0.623
2024-25	Harvest	Control	Fruit	Fructose	6	16.43	1.064	0.434
2024-25	Harvest	Defoliated	Fruit	Fructose	3	15.23	1.411	0.815
2023-24	1 day after defoliation	Control	Fruit	Glucose	6	10.99	1.473	0.602
2023-24	1 day after defoliation	Defoliated	Fruit	Glucose	6	10.50	1.370	0.559
2023-24	15 days after defoliation	Control	Fruit	Glucose	6	11.55	1.635	0.668
2023-24	15 days after defoliation	Defoliated	Fruit	Glucose	6	12.46	1.355	0.553
2023-24	36 days after defoliation	Control	Fruit	Glucose	6	10.50	1.198	0.489
2023-24	36 days after defoliation	Defoliated	Fruit	Glucose	6	11.26	1.176	0.480
2023-24	64 days after defoliation	Control	Fruit	Glucose	6	7.29	0.848	0.346
2023-24	64 days after defoliation	Defoliated	Fruit	Glucose	6	10.58	1.722	0.703
2024-25	Early fruit growth	Control	Fruit	Glucose	6	1.85	0.630	0.257
2024-25	Early fruit growth	Defoliated	Fruit	Glucose	3	1.06	0.470	0.271
2024-25	Harvest	Control	Fruit	Glucose	6	5.80	1.315	0.537
2024-25	Harvest	Defoliated	Fruit	Glucose	3	5.43	0.317	0.183
2023-24	1 day after defoliation	Control	Fruit	Sucrose	6	14.23	1.170	0.478
2023-24	1 day after defoliation	Defoliated	Fruit	Sucrose	6	12.45	1.820	0.743
2023-24	15 days after defoliation	Control	Fruit	Sucrose	6	13.74	2.714	1.108
2023-24	15 days after defoliation	Defoliated	Fruit	Sucrose	6	14.43	2.350	0.959
2023-24	36 days after defoliation	Control	Fruit	Sucrose	6	9.78	2.167	0.885
2023-24	36 days after defoliation	Defoliated	Fruit	Sucrose	6	12.58	1.114	0.455
2023-24	64 days after defoliation	Control	Fruit	Sucrose	6	7.36	2.542	1.038
2023-24	64 days after defoliation	Defoliated	Fruit	Sucrose	6	11.63	1.792	0.732
2024-25	Early fruit growth	Control	Fruit	Sucrose	6	4.46	1.631	0.666
2024-25	Early fruit growth	Defoliated	Fruit	Sucrose	3	5.64	1.195	0.690
2024-25	Harvest	Control	Fruit	Sucrose	6	12.71	1.480	0.604
2024-25	Harvest	Defoliated	Fruit	Sucrose	3	13.96	1.686	0.973

Season	Sampling time	Treatment	Organ	Sugar	n	Mean	Standard Deviation	Standard Error
2023-24	1 day after defoliation	Control	Medium roots	Fructose	6	0.41	0.195	0.080
2023-24	1 day after defoliation	Defoliated	Medium roots	Fructose	6	0.37	0.214	0.087
2023-24	15 days after defoliation	Control	Medium roots	Fructose	6	0.35	0.053	0.022
2023-24	15 days after defoliation	Defoliated	Medium roots	Fructose	6	0.33	0.176	0.072
2023-24	36 days after defoliation	Control	Medium roots	Fructose	6	0.25	0.085	0.035
2023-24	36 days after defoliation	Defoliated	Medium roots	Fructose	6	0.24	0.096	0.039
2023-24	64 days after defoliation	Control	Medium roots	Fructose	6	0.30	0.093	0.038
2023-24	64 days after defoliation	Defoliated	Medium roots	Fructose	6	0.21	0.053	0.022
2024-25	Vegetative development	Control	Medium roots	Fructose	6	0.46	0.183	0.075
2024-25	Vegetative development	Defoliated	Medium roots	Fructose	6	0.28	0.209	0.086
2024-25	Panicle emergence	Control	Medium roots	Fructose	6	0.34	0.109	0.044
2024-25	Panicle emergence	Defoliated	Medium roots	Fructose	6	0.34	0.097	0.040
2024-25	Early fruit growth	Control	Medium roots	Fructose	6	0.45	0.054	0.022
2024-25	Early fruit growth	Defoliated	Medium roots	Fructose	6	0.38	0.110	0.045
2024-25	Harvest	Control	Medium roots	Fructose	6	0.46	0.109	0.045
2024-25	Harvest	Defoliated	Medium roots	Fructose	6	0.47	0.199	0.081
2023-24	1 day after defoliation	Control	Medium roots	Glucose	6	0.15	0.129	0.053
2023-24	1 day after defoliation	Defoliated	Medium roots	Glucose	6	0.39	0.392	0.160
2023-24	15 days after defoliation	Control	Medium roots	Glucose	6	0.40	0.184	0.075
2023-24	15 days after defoliation	Defoliated	Medium roots	Glucose	6	0.12	0.077	0.032
2023-24	36 days after defoliation	Control	Medium roots	Glucose	6	0.04	0.048	0.020
2023-24	36 days after defoliation	Defoliated	Medium roots	Glucose	6	0.23	0.152	0.062
2023-24	64 days after defoliation	Control	Medium roots	Glucose	6	0.07	0.064	0.026
2023-24	64 days after defoliation	Defoliated	Medium roots	Glucose	6	0.17	0.237	0.097
2024-25	Vegetative development	Control	Medium roots	Glucose	6	0.51	0.177	0.072
2024-25	Vegetative development	Defoliated	Medium roots	Glucose	6	0.51	0.217	0.089
2024-25	Panicle emergence	Control	Medium roots	Glucose	6	0.51	0.088	0.036
2024-25	Panicle emergence	Defoliated	Medium roots	Glucose	6	0.55	0.139	0.057
2024-25	Early fruit growth	Control	Medium roots	Glucose	6	0.49	0.064	0.026
2024-25	Early fruit growth	Defoliated	Medium roots	Glucose	6	0.58	0.194	0.079
2024-25	Harvest	Control	Medium roots	Glucose	6	0.55	0.130	0.053
2024-25	Harvest	Defoliated	Medium roots	Glucose	6	0.55	0.178	0.073
2023-24	1 day after defoliation	Control	Medium roots	Sucrose	6	2.04	0.447	0.183
2023-24	1 day after defoliation	Defoliated	Medium roots	Sucrose	6	1.89	0.648	0.265
2023-24	15 days after defoliation	Control	Medium roots	Sucrose	6	1.72	0.510	0.208
2023-24	15 days after defoliation	Defoliated	Medium roots	Sucrose	6	1.73	0.274	0.112
2023-24	36 days after defoliation	Control	Medium roots	Sucrose	6	1.45	0.470	0.192
2023-24	36 days after defoliation	Defoliated	Medium roots	Sucrose	6	1.06	0.515	0.210
2023-24	64 days after defoliation	Control	Medium roots	Sucrose	6	1.50	0.731	0.298
2023-24	64 days after defoliation	Defoliated	Medium roots	Sucrose	6	1.30	0.535	0.219
2024-25	Vegetative development	Control	Medium roots	Sucrose	6	2.09	0.519	0.212
2024-25	Vegetative development	Defoliated	Medium roots	Sucrose	6	1.96	0.884	0.361
2024-25	Panicle emergence	Control	Medium roots	Sucrose	6	3.57	0.633	0.258
2024-25	Panicle emergence	Defoliated	Medium roots	Sucrose	6	3.91	1.287	0.525
2024-25	Early fruit growth	Control	Medium roots	Sucrose	6	3.15	0.737	0.301
2024-25	Early fruit growth	Defoliated	Medium roots	Sucrose	6	3.62	0.718	0.293
2024-25	Harvest	Control	Medium roots	Sucrose	6	2.31	1.583	0.646
2024-25	Harvest	Defoliated	Medium roots	Sucrose	6	2.72	2.013	0.822

Season	Sampling time	Treatment	Organ	Sugar	n	Mean	Standard Deviation	Standard Error
2024-25	Vegetative development	Control	Fine roots	Fructose	6	0.61	0.100	0.041
2024-25	Vegetative development	Defoliated	Fine roots	Fructose	6	0.50	0.095	0.039
2024-25	Panicle emergence	Control	Fine roots	Fructose	6	0.49	0.142	0.058
2024-25	Panicle emergence	Defoliated	Fine roots	Fructose	6	0.40	0.086	0.035
2024-25	Early fruit growth	Control	Fine roots	Fructose	6	0.43	0.057	0.023
2024-25	Early fruit growth	Defoliated	Fine roots	Fructose	6	0.32	0.109	0.044
2024-25	Harvest	Control	Fine roots	Fructose	6	0.48	0.239	0.097
2024-25	Harvest	Defoliated	Fine roots	Fructose	6	0.55	0.150	0.061
2024-25	Vegetative development	Control	Fine roots	Glucose	6	0.53	0.080	0.033
2024-25	Vegetative development	Defoliated	Fine roots	Glucose	6	0.73	0.220	0.090
2024-25	Panicle emergence	Control	Fine roots	Glucose	6	0.64	0.166	0.068
2024-25	Panicle emergence	Defoliated	Fine roots	Glucose	6	0.64	0.083	0.034
2024-25	Early fruit growth	Control	Fine roots	Glucose	6	0.59	0.072	0.029
2024-25	Early fruit growth	Defoliated	Fine roots	Glucose	6	0.52	0.110	0.045
2024-25	Harvest	Control	Fine roots	Glucose	6	0.69	0.348	0.142
2024-25	Harvest	Defoliated	Fine roots	Glucose	6	0.49	0.156	0.064
2024-25	Vegetative development	Control	Fine roots	Sucrose	6	2.79	0.193	0.079
2024-25	Vegetative development	Defoliated	Fine roots	Sucrose	6	3.18	0.835	0.341
2024-25	Panicle emergence	Control	Fine roots	Sucrose	6	3.57	0.793	0.324
2024-25	Panicle emergence	Defoliated	Fine roots	Sucrose	6	3.95	0.202	0.082
2024-25	Early fruit growth	Control	Fine roots	Sucrose	6	3.32	0.493	0.201
2024-25	Early fruit growth	Defoliated	Fine roots	Sucrose	6	3.71	0.631	0.257
2024-25	Harvest	Control	Fine roots	Sucrose	6	3.06	1.932	0.789
2024-25	Harvest	Defoliated	Fine roots	Sucrose	6	3.23	0.464	0.190
2024-25	Vegetative development	Control	coarse roots	Fructose	6	0.62	0.095	0.039
2024-25	Vegetative development	Defoliated	coarse roots	Fructose	6	0.91	0.277	0.113
2024-25	Panicle emergence	Control	coarse roots	Fructose	6	0.26	0.206	0.084
2024-25	Panicle emergence	Defoliated	coarse roots	Fructose	6	0.48	0.139	0.057
2024-25	Early fruit growth	Control	coarse roots	Fructose	6	0.18	0.063	0.026
2024-25	Early fruit growth	Defoliated	coarse roots	Fructose	6	0.30	0.088	0.036
2024-25	Harvest	Control	coarse roots	Fructose	6	0.53	0.409	0.167
2024-25	Harvest	Defoliated	coarse roots	Fructose	6	0.34	0.227	0.093
2024-25	Vegetative development	Control	coarse roots	Glucose	6	0.72	0.166	0.068
2024-25	Vegetative development	Defoliated	coarse roots	Glucose	6	1.29	0.354	0.145
2024-25	Panicle emergence	Control	coarse roots	Glucose	6	0.37	0.092	0.037
2024-25	Panicle emergence	Defoliated	coarse roots	Glucose	6	0.78	0.239	0.098
2024-25	Early fruit growth	Control	coarse roots	Glucose	6	0.39	0.158	0.065
2024-25	Early fruit growth	Defoliated	coarse roots	Glucose	6	0.56	0.189	0.077
2024-25	Harvest	Control	coarse roots	Glucose	6	0.71	0.486	0.199
2024-25	Harvest	Defoliated	coarse roots	Glucose	6	0.37	0.134	0.055
2024-25	Vegetative development	Control	coarse roots	Sucrose	6	2.63	0.686	0.280
2024-25	Vegetative development	Defoliated	coarse roots	Sucrose	6	3.65	1.017	0.415
2024-25	Panicle emergence	Control	coarse roots	Sucrose	6	2.26	0.498	0.203
2024-25	Panicle emergence	Defoliated	coarse roots	Sucrose	6	3.42	0.504	0.206
2024-25	Early fruit growth	Control	coarse roots	Sucrose	6	1.53	0.362	0.148
2024-25	Early fruit growth	Defoliated	coarse roots	Sucrose	6	2.28	0.724	0.296
2024-25	Harvest	Control	coarse roots	Sucrose	6	1.99	0.506	0.207
2024-25	Harvest	Defoliated	coarse roots	Sucrose	6	2.53	1.024	0.418

Season	Sampling time	Treatment	Organ	Sugar	n	Mean	Standard Deviation	Standard Error
2023-24	1 day after defoliation	Control	Trunks	Fructose	6	0.08	0.060	0.024
2023-24	1 day after defoliation	Defoliated	Trunks	Fructose	6	0.16	0.064	0.026
2023-24	15 days after defoliation	Control	Trunks	Fructose	6	0.20	0.083	0.034
2023-24	15 days after defoliation	Defoliated	Trunks	Fructose	6	0.23	0.066	0.027
2023-24	36 days after defoliation	Control	Trunks	Fructose	6	0.33	0.115	0.047
2023-24	36 days after defoliation	Defoliated	Trunks	Fructose	6	0.33	0.062	0.025
2023-24	64 days after defoliation	Control	Trunks	Fructose	6	0.31	0.109	0.045
2023-24	64 days after defoliation	Defoliated	Trunks	Fructose	6	0.41	0.065	0.026
2024-25	Vegetative development	Control	Trunks	Fructose	6	0.48	0.166	0.068
2024-25	Vegetative development	Defoliated	Trunks	Fructose	6	0.52	0.144	0.059
2024-25	Panicle emergence	Control	Trunks	Fructose	6	0.23	0.064	0.026
2024-25	Panicle emergence	Defoliated	Trunks	Fructose	6	0.63	0.485	0.198
2024-25	Early fruit growth	Control	Trunks	Fructose	6	0.20	0.061	0.025
2024-25	Early fruit growth	Defoliated	Trunks	Fructose	6	0.25	0.059	0.024
2024-25	Harvest	Control	Trunks	Fructose	6	0.24	0.083	0.034
2024-25	Harvest	Defoliated	Trunks	Fructose	6	0.14	0.072	0.030
2023-24	1 day after defoliation	Control	Trunks	Glucose	6	0.14	0.060	0.025
2023-24	1 day after defoliation	Defoliated	Trunks	Glucose	6	0.13	0.039	0.016
2023-24	15 days after defoliation	Control	Trunks	Glucose	6	0.20	0.068	0.028
2023-24	15 days after defoliation	Defoliated	Trunks	Glucose	6	0.22	0.042	0.017
2023-24	36 days after defoliation	Control	Trunks	Glucose	6	0.28	0.080	0.033
2023-24	36 days after defoliation	Defoliated	Trunks	Glucose	6	0.32	0.085	0.035
2023-24	64 days after defoliation	Control	Trunks	Glucose	6	0.28	0.054	0.022
2023-24	64 days after defoliation	Defoliated	Trunks	Glucose	6	0.42	0.030	0.012
2024-25	Vegetative development	Control	Trunks	Glucose	6	0.32	0.135	0.055
2024-25	Vegetative development	Defoliated	Trunks	Glucose	6	0.46	0.176	0.072
2024-25	Panicle emergence	Control	Trunks	Glucose	6	0.21	0.063	0.026
2024-25	Panicle emergence	Defoliated	Trunks	Glucose	6	0.53	0.425	0.173
2024-25	Early fruit growth	Control	Trunks	Glucose	6	0.23	0.043	0.018
2024-25	Early fruit growth	Defoliated	Trunks	Glucose	6	0.28	0.031	0.013
2024-25	Harvest	Control	Trunks	Glucose	6	0.32	0.095	0.039
2024-25	Harvest	Defoliated	Trunks	Glucose	6	0.25	0.038	0.016
2023-24	1 day after defoliation	Control	Trunks	Sucrose	6	1.33	0.331	0.135
2023-24	1 day after defoliation	Defoliated	Trunks	Sucrose	6	1.30	0.351	0.143
2023-24	15 days after defoliation	Control	Trunks	Sucrose	6	1.95	0.360	0.147
2023-24	15 days after defoliation	Defoliated	Trunks	Sucrose	6	1.76	0.241	0.099
2023-24	36 days after defoliation	Control	Trunks	Sucrose	6	2.03	0.472	0.193
2023-24	36 days after defoliation	Defoliated	Trunks	Sucrose	6	1.78	0.300	0.122
2023-24	64 days after defoliation	Control	Trunks	Sucrose	6	2.10	0.200	0.082
2023-24	64 days after defoliation	Defoliated	Trunks	Sucrose	6	1.51	0.321	0.131
2024-25	Vegetative development	Control	Trunks	Sucrose	6	2.24	0.657	0.268
2024-25	Vegetative development	Defoliated	Trunks	Sucrose	6	1.63	0.422	0.172
2024-25	Panicle emergence	Control	Trunks	Sucrose	6	2.09	0.307	0.125
2024-25	Panicle emergence	Defoliated	Trunks	Sucrose	6	2.52	0.754	0.308
2024-25	Early fruit growth	Control	Trunks	Sucrose	6	1.31	0.253	0.103
2024-25	Early fruit growth	Defoliated	Trunks	Sucrose	6	1.59	0.337	0.138
2024-25	Harvest	Control	Trunks	Sucrose	6	1.49	0.584	0.239
2024-25	Harvest	Defoliated	Trunks	Sucrose	6	2.03	0.301	0.123

Season	Sampling time	Treatment	Organ	Sugar	n	Mean	Standard Deviation	Standard Error
2024-25	Vegetative development	Control	Primary branches	Fructose	6	0.65	0.224	0.091
2024-25	Vegetative development	Defoliated	Primary branches	Fructose	6	0.49	0.232	0.095
2024-25	Panicle emergence	Control	Primary branches	Fructose	6	0.30	0.127	0.052
2024-25	Panicle emergence	Defoliated	Primary branches	Fructose	6	0.42	0.067	0.028
2024-25	Early fruit growth	Control	Primary branches	Fructose	6	0.52	0.202	0.083
2024-25	Early fruit growth	Defoliated	Primary branches	Fructose	6	0.51	0.264	0.108
2024-25	Harvest	Control	Primary branches	Fructose	6	0.44	0.097	0.040
2024-25	Harvest	Defoliated	Primary branches	Fructose	6	0.29	0.145	0.059
2024-25	Vegetative development	Control	Primary branches	Glucose	6	0.35	0.312	0.127
2024-25	Vegetative development	Defoliated	Primary branches	Glucose	6	0.48	0.191	0.078
2024-25	Panicle emergence	Control	Primary branches	Glucose	6	0.32	0.129	0.053
2024-25	Panicle emergence	Defoliated	Primary branches	Glucose	6	0.33	0.126	0.052
2024-25	Early fruit growth	Control	Primary branches	Glucose	6	0.49	0.193	0.079
2024-25	Early fruit growth	Defoliated	Primary branches	Glucose	6	0.48	0.185	0.075
2024-25	Harvest	Control	Primary branches	Glucose	6	0.44	0.118	0.048
2024-25	Harvest	Defoliated	Primary branches	Glucose	6	0.36	0.142	0.058
2024-25	Vegetative development	Control	Primary branches	Sucrose	6	2.38	0.680	0.278
2024-25	Vegetative development	Defoliated	Primary branches	Sucrose	6	1.69	0.362	0.148
2024-25	Panicle emergence	Control	Primary branches	Sucrose	6	1.92	0.677	0.276
2024-25	Panicle emergence	Defoliated	Primary branches	Sucrose	6	2.41	0.342	0.140
2024-25	Early fruit growth	Control	Primary branches	Sucrose	6	2.39	0.524	0.214
2024-25	Early fruit growth	Defoliated	Primary branches	Sucrose	6	2.37	0.752	0.307
2024-25	Harvest	Control	Primary branches	Sucrose	6	1.85	0.999	0.408
2024-25	Harvest	Defoliated	Primary branches	Sucrose	6	2.36	0.150	0.061
2024-25	Vegetative development	Control	Tertiary branches	Fructose	6	0.56	0.192	0.078
2024-25	Vegetative development	Defoliated	Tertiary branches	Fructose	6	0.44	0.066	0.027
2024-25	Panicle emergence	Control	Tertiary branches	Fructose	6	0.28	0.052	0.021
2024-25	Panicle emergence	Defoliated	Tertiary branches	Fructose	6	0.38	0.075	0.030
2024-25	Early fruit growth	Control	Tertiary branches	Fructose	6	0.38	0.094	0.039
2024-25	Early fruit growth	Defoliated	Tertiary branches	Fructose	6	0.33	0.177	0.072
2024-25	Harvest	Control	Tertiary branches	Fructose	6	0.28	0.102	0.042
2024-25	Harvest	Defoliated	Tertiary branches	Fructose	6	0.28	0.252	0.103
2024-25	Vegetative development	Control	Tertiary branches	Glucose	6	0.43	0.209	0.086
2024-25	Vegetative development	Defoliated	Tertiary branches	Glucose	6	0.30	0.077	0.032
2024-25	Panicle emergence	Control	Tertiary branches	Glucose	6	0.22	0.044	0.018
2024-25	Panicle emergence	Defoliated	Tertiary branches	Glucose	6	0.27	0.097	0.040
2024-25	Early fruit growth	Control	Tertiary branches	Glucose	6	0.40	0.076	0.031
2024-25	Early fruit growth	Defoliated	Tertiary branches	Glucose	6	0.53	0.310	0.127
2024-25	Harvest	Control	Tertiary branches	Glucose	6	0.36	0.079	0.032
2024-25	Harvest	Defoliated	Tertiary branches	Glucose	6	0.30	0.105	0.043
2024-25	Vegetative development	Control	Tertiary branches	Sucrose	6	2.07	0.583	0.238
2024-25	Vegetative development	Defoliated	Tertiary branches	Sucrose	6	1.26	0.515	0.210
2024-25	Panicle emergence	Control	Tertiary branches	Sucrose	6	1.90	0.343	0.140
2024-25	Panicle emergence	Defoliated	Tertiary branches	Sucrose	6	1.90	0.544	0.222
2024-25	Early fruit growth	Control	Tertiary branches	Sucrose	6	2.06	0.388	0.158
2024-25	Early fruit growth	Defoliated	Tertiary branches	Sucrose	6	1.83	0.579	0.236
2024-25	Harvest	Control	Tertiary branches	Sucrose	6	1.75	0.791	0.323
2024-25	Harvest	Defoliated	Tertiary branches	Sucrose	6	1.92	0.769	0.314

Season	Sampling time	Treatment	Organ	Sugar	n	Mean	Standard Deviation	Standard Error
2023-24	1 day after defoliation	Control	Fruiting shoots	Fructose	6	0.89	0.144	0.059
2023-24	1 day after defoliation	Defoliated	Fruiting shoots	Fructose	6	1.01	0.138	0.057
2023-24	15 days after defoliation	Control	Fruiting shoots	Fructose	6	1.10	0.264	0.108
2023-24	15 days after defoliation	Defoliated	Fruiting shoots	Fructose	6	0.67	0.250	0.102
2023-24	36 days after defoliation	Control	Fruiting shoots	Fructose	6	1.23	0.137	0.056
2023-24	36 days after defoliation	Defoliated	Fruiting shoots	Fructose	6	0.52	0.215	0.088
2023-24	64 days after defoliation	Control	Fruiting shoots	Fructose	6	1.03	0.223	0.091
2023-24	64 days after defoliation	Defoliated	Fruiting shoots	Fructose	6	0.37	0.085	0.035
2024-25	Panicle emergence	Control	Fruiting shoots	Fructose	6	0.50	0.090	0.037
2024-25	Panicle emergence	Defoliated	Fruiting shoots	Fructose	6	0.52	0.152	0.062
2024-25	Early fruit growth	Control	Fruiting shoots	Fructose	6	0.73	0.167	0.068
2024-25	Early fruit growth	Defoliated	Fruiting shoots	Fructose	3	0.82	0.178	0.103
2024-25	Harvest	Control	Fruiting shoots	Fructose	6	0.76	0.228	0.093
2024-25	Harvest	Defoliated	Fruiting shoots	Fructose	3	0.80	0.160	0.092
2023-24	1 day after defoliation	Control	Fruiting shoots	Glucose	6	0.51	0.141	0.057
2023-24	1 day after defoliation	Defoliated	Fruiting shoots	Glucose	6	0.76	0.678	0.277
2023-24	15 days after defoliation	Control	Fruiting shoots	Glucose	6	0.68	0.197	0.080
2023-24	15 days after defoliation	Defoliated	Fruiting shoots	Glucose	6	0.30	0.184	0.075
2023-24	36 days after defoliation	Control	Fruiting shoots	Glucose	6	0.95	0.236	0.097
2023-24	36 days after defoliation	Defoliated	Fruiting shoots	Glucose	6	0.14	0.129	0.053
2023-24	64 days after defoliation	Control	Fruiting shoots	Glucose	6	0.76	0.060	0.025
2023-24	64 days after defoliation	Defoliated	Fruiting shoots	Glucose	6	0.06	0.057	0.023
2024-25	Panicle emergence	Control	Fruiting shoots	Glucose	6	0.63	0.132	0.054
2024-25	Panicle emergence	Defoliated	Fruiting shoots	Glucose	6	0.45	0.094	0.038
2024-25	Early fruit growth	Control	Fruiting shoots	Glucose	6	0.82	0.187	0.076
2024-25	Early fruit growth	Defoliated	Fruiting shoots	Glucose	3	0.87	0.085	0.049
2024-25	Harvest	Control	Fruiting shoots	Glucose	6	0.84	0.263	0.107
2024-25	Harvest	Defoliated	Fruiting shoots	Glucose	3	0.89	0.036	0.021
2023-24	1 day after defoliation	Control	Fruiting shoots	Sucrose	6	5.57	1.333	0.544
2023-24	1 day after defoliation	Defoliated	Fruiting shoots	Sucrose	6	4.33	0.888	0.362
2023-24	15 days after defoliation	Control	Fruiting shoots	Sucrose	6	4.67	1.250	0.510
2023-24	15 days after defoliation	Defoliated	Fruiting shoots	Sucrose	5	2.20	0.596	0.266
2023-24	36 days after defoliation	Control	Fruiting shoots	Sucrose	6	4.26	0.957	0.391
2023-24	36 days after defoliation	Defoliated	Fruiting shoots	Sucrose	6	1.91	0.779	0.318
2023-24	64 days after defoliation	Control	Fruiting shoots	Sucrose	6	4.54	0.871	0.356
2023-24	64 days after defoliation	Defoliated	Fruiting shoots	Sucrose	6	1.08	0.179	0.073
2024-25	Panicle emergence	Control	Fruiting shoots	Sucrose	6	5.16	0.941	0.384
2024-25	Panicle emergence	Defoliated	Fruiting shoots	Sucrose	6	3.95	0.604	0.247
2024-25	Early fruit growth	Control	Fruiting shoots	Sucrose	6	5.19	1.024	0.418
2024-25	Early fruit growth	Defoliated	Fruiting shoots	Sucrose	3	6.29	0.207	0.119
2024-25	Harvest	Control	Fruiting shoots	Sucrose	6	4.95	1.397	0.570
2024-25	Harvest	Defoliated	Fruiting shoots	Sucrose	3	5.42	0.801	0.463
2023-24	64 days after defoliation	Control	Non-fruiting shoots	Fructose	6	1.14	0.187	0.076
2023-24	64 days after defoliation	Defoliated	Non-fruiting shoots	Fructose	6	0.53	0.229	0.093
2024-25	Early fruit growth	Control	Non-fruiting shoots	Fructose	6	0.88	0.144	0.059
2024-25	Early fruit growth	Defoliated	Non-fruiting shoots	Fructose	6	0.90	0.174	0.071
2024-25	Harvest	Control	Non-fruiting shoots	Fructose	6	0.68	0.199	0.081
2024-25	Harvest	Defoliated	Non-fruiting shoots	Fructose	6	0.66	0.226	0.092
2023-24	64 days after defoliation	Control	Non-fruiting shoots	Glucose	6	0.76	0.167	0.068
2023-24	64 days after defoliation	Defoliated	Non-fruiting shoots	Glucose	6	0.26	0.163	0.067
2024-25	Early fruit growth	Control	Non-fruiting shoots	Glucose	6	1.00	0.410	0.168
2024-25	Early fruit growth	Defoliated	Non-fruiting shoots	Glucose	6	0.95	0.134	0.055
2024-25	Harvest	Control	Non-fruiting shoots	Glucose	6	0.85	0.344	0.140
2024-25	Harvest	Defoliated	Non-fruiting shoots	Glucose	6	0.63	0.186	0.076
2023-24	64 days after defoliation	Control	Non-fruiting shoots	Sucrose	6	4.87	1.004	0.410
2023-24	64 days after defoliation	Defoliated	Non-fruiting shoots	Sucrose	6	1.16	0.363	0.148
2024-25	Early fruit growth	Control	Non-fruiting shoots	Sucrose	6	5.63	0.748	0.306
2024-25	Early fruit growth	Defoliated	Non-fruiting shoots	Sucrose	6	4.94	0.610	0.249
2024-25	Harvest	Control	Non-fruiting shoots	Sucrose	6	4.27	0.911	0.372
2024-25	Harvest	Defoliated	Non-fruiting shoots	Sucrose	6	4.25	0.778	0.318

Season	Sampling time	Treatment	Organ	Sugar	n	Mean	Standard Deviation	Standard Error
2023-24	1 day after defoliation	Control	Pedicels	Fructose	6	0.93	0.151	0.062
2023-24	1 day after defoliation	Defoliated	Pedicels	Fructose	6	1.19	0.092	0.038
2023-24	15 days after defoliation	Control	Pedicels	Fructose	6	1.68	0.305	0.124
2023-24	15 days after defoliation	Defoliated	Pedicels	Fructose	6	1.30	0.221	0.090
2023-24	36 days after defoliation	Control	Pedicels	Fructose	6	1.40	0.272	0.111
2023-24	36 days after defoliation	Defoliated	Pedicels	Fructose	6	1.58	0.728	0.297
2023-24	64 days after defoliation	Control	Pedicels	Fructose	6	1.61	0.116	0.047
2023-24	64 days after defoliation	Defoliated	Pedicels	Fructose	6	1.18	0.291	0.119
2024-25	Harvest	Control	Pedicels	Fructose	6	1.21	0.145	0.059
2024-25	Harvest	Defoliated	Pedicels	Fructose	3	1.71	0.143	0.082
2023-24	1 day after defoliation	Control	Pedicels	Glucose	6	0.95	0.214	0.088
2023-24	1 day after defoliation	Defoliated	Pedicels	Glucose	6	1.27	0.136	0.056
2023-24	15 days after defoliation	Control	Pedicels	Glucose	6	1.58	0.643	0.263
2023-24	15 days after defoliation	Defoliated	Pedicels	Glucose	6	1.39	0.308	0.126
2023-24	36 days after defoliation	Control	Pedicels	Glucose	6	1.49	0.426	0.174
2023-24	36 days after defoliation	Defoliated	Pedicels	Glucose	6	1.90	1.306	0.533
2023-24	64 days after defoliation	Control	Pedicels	Glucose	6	1.63	0.634	0.259
2023-24	64 days after defoliation	Defoliated	Pedicels	Glucose	6	1.19	0.328	0.134
2024-25	Harvest	Control	Pedicels	Glucose	6	1.44	0.198	0.081
2024-25	Harvest	Defoliated	Pedicels	Glucose	3	1.64	0.164	0.095
2023-24	1 day after defoliation	Control	Pedicels	Sucrose	6	5.25	0.609	0.249
2023-24	1 day after defoliation	Defoliated	Pedicels	Sucrose	6	4.21	1.059	0.432
2023-24	15 days after defoliation	Control	Pedicels	Sucrose	6	5.86	1.421	0.580
2023-24	15 days after defoliation	Defoliated	Pedicels	Sucrose	6	3.06	1.048	0.428
2023-24	36 days after defoliation	Control	Pedicels	Sucrose	6	3.64	1.297	0.530
2023-24	36 days after defoliation	Defoliated	Pedicels	Sucrose	6	2.09	1.475	0.602
2023-24	64 days after defoliation	Control	Pedicels	Sucrose	6	4.82	1.071	0.437
2023-24	64 days after defoliation	Defoliated	Pedicels	Sucrose	6	1.93	0.817	0.333
2024-25	Harvest	Control	Pedicels	Sucrose	6	8.79	0.668	0.273
2024-25	Harvest	Defoliated	Pedicels	Sucrose	3	8.99	0.481	0.278

Season	Sampling time	Treatment	Organ	Sugar	n	Mean	Standard Deviation	Standard Error
2023-24	1 day after defoliation	Control	Leaves	Fructose	6	0.59	0.253	0.103
2023-24	1 day after defoliation	Defoliated	Leaves	Fructose	6	0.48	0.172	0.070
2023-24	15 days after defoliation	Control	Leaves	Fructose	6	0.38	0.154	0.063
2023-24	15 days after defoliation	Defoliated	Leaves	Fructose	6	0.19	0.031	0.013
2023-24	36 days after defoliation	Control	Leaves	Fructose	6	0.45	0.093	0.038
2023-24	36 days after defoliation	Defoliated	Leaves	Fructose	6	0.17	0.067	0.027
2023-24	64 days after defoliation	Control	Leaves	Fructose	6	0.59	0.196	0.080
2023-24	64 days after defoliation	Defoliated	Leaves	Fructose	6	0.36	0.152	0.062
2024-25	Vegetative development	Control	Leaves	Fructose	6	0.39	0.115	0.047
2024-25	Vegetative development	Defoliated	Leaves	Fructose	6	0.45	0.080	0.033
2024-25	Panicle emergence	Control	Leaves	Fructose	6	0.34	0.076	0.031
2024-25	Panicle emergence	Defoliated	Leaves	Fructose	6	0.37	0.091	0.037
2024-25	Early fruit growth	Control	Leaves	Fructose	6	0.55	0.117	0.048
2024-25	Early fruit growth	Defoliated	Leaves	Fructose	6	0.46	0.165	0.067
2024-25	Harvest	Control	Leaves	Fructose	6	0.38	0.191	0.078
2024-25	Harvest	Defoliated	Leaves	Fructose	6	0.39	0.132	0.054
2023-24	1 day after defoliation	Control	Leaves	Glucose	6	0.56	0.180	0.074
2023-24	1 day after defoliation	Defoliated	Leaves	Glucose	6	0.42	0.090	0.037
2023-24	15 days after defoliation	Control	Leaves	Glucose	6	0.61	0.597	0.244
2023-24	15 days after defoliation	Defoliated	Leaves	Glucose	6	0.26	0.030	0.012
2023-24	36 days after defoliation	Control	Leaves	Glucose	6	0.49	0.066	0.027
2023-24	36 days after defoliation	Defoliated	Leaves	Glucose	6	0.24	0.070	0.029
2023-24	64 days after defoliation	Control	Leaves	Glucose	6	0.55	0.145	0.059
2023-24	64 days after defoliation	Defoliated	Leaves	Glucose	6	0.38	0.142	0.058
2024-25	Vegetative development	Control	Leaves	Glucose	6	0.46	0.114	0.047
2024-25	Vegetative development	Defoliated	Leaves	Glucose	6	0.51	0.069	0.028
2024-25	Panicle emergence	Control	Leaves	Glucose	6	0.33	0.072	0.030
2024-25	Panicle emergence	Defoliated	Leaves	Glucose	6	0.34	0.050	0.020
2024-25	Early fruit growth	Control	Leaves	Glucose	6	0.68	0.094	0.038
2024-25	Early fruit growth	Defoliated	Leaves	Glucose	6	0.61	0.128	0.052
2024-25	Harvest	Control	Leaves	Glucose	6	0.43	0.139	0.057
2024-25	Harvest	Defoliated	Leaves	Glucose	6	0.44	0.117	0.048
2023-24	1 day after defoliation	Control	Leaves	Sucrose	6	5.75	0.529	0.216
2023-24	1 day after defoliation	Defoliated	Leaves	Sucrose	6	5.92	0.385	0.157
2023-24	15 days after defoliation	Control	Leaves	Sucrose	6	5.30	1.154	0.471
2023-24	15 days after defoliation	Defoliated	Leaves	Sucrose	6	5.82	0.188	0.077
2023-24	36 days after defoliation	Control	Leaves	Sucrose	6	6.08	0.578	0.236
2023-24	36 days after defoliation	Defoliated	Leaves	Sucrose	6	6.58	0.506	0.207
2023-24	64 days after defoliation	Control	Leaves	Sucrose	6	5.91	0.649	0.265
2023-24	64 days after defoliation	Defoliated	Leaves	Sucrose	6	9.10	0.992	0.405
2024-25	Vegetative development	Control	Leaves	Sucrose	6	9.17	0.810	0.331
2024-25	Vegetative development	Defoliated	Leaves	Sucrose	6	9.56	0.950	0.388
2024-25	Panicle emergence	Control	Leaves	Sucrose	6	8.06	0.590	0.241
2024-25	Panicle emergence	Defoliated	Leaves	Sucrose	6	7.78	1.163	0.475
2024-25	Early fruit growth	Control	Leaves	Sucrose	6	5.85	0.491	0.200
2024-25	Early fruit growth	Defoliated	Leaves	Sucrose	6	5.90	0.474	0.193
2024-25	Harvest	Control	Leaves	Sucrose	6	5.76	0.874	0.357
2024-25	Harvest	Defoliated	Leaves	Sucrose	6	5.81	1.069	0.436

Supplementary table S2: Spearman rank correlation coefficients and associated *P*-values for seasonal changes in total non-structural carbohydrate concentration (ΔNSC) among organs. ΔNSC was calculated as the difference between final and first sampling dates within each season.

2023-2024					
**Correlation coefficient**	Medium roots ΔNSC	Trunks ΔNSC	Shoots ΔNSC	Pedicels ΔNSC	Leaves ΔNSC
Medium roots ΔNSC	1	0.238	-0.357	0.294	0.049
Trunks ΔNSC	0.238	1	0.552	0.678	-0.594
Shoots ΔNSC	-0.357	0.552	1	0.420	-0.622
Pedicels ΔNSC	0.294	0.678	0.420	1	-0.867
Leaves ΔNSC	0.049	-0.594	-0.622	-0.867	1
**P-value**	Medium roots ΔNSC	Trunks ΔNSC	Shoots ΔNSC	Pedicels ΔNSC	Leaves ΔNSC
Medium roots ΔNSC	1	0.457	0.255	0.354	0.880
Trunks ΔNSC	0.457	1	0.063	0.015	0.042
Shoots ΔNSC	0.255	0.063	1	0.175	0.031
Pedicels ΔNSC	0.354	0.015	0.175	1	0.000
Leaves ΔNSC	0.880	0.042	0.031	0.000	1

2024-2025							
**Correlation coefficient**	Medium roots ΔNSC	Coarse roots ΔNSC	Fine roots ΔNSC	Trunks ΔNSC	Primary branches ΔNSC	Tertiary branches ΔNSC	Leaves ΔNSC
Medium roots ΔNSC	1	0.315	0.538	0.531	0.322	0.720	0.601
Coarse roots ΔNSC	0.315	1	0.133	0.678	0.490	0.650	0.161
Fine roots ΔNSC	0.538	0.133	1	0.371	0.329	0.552	0.287
Trunks ΔNSC	0.531	0.678	0.371	1	0.797	0.902	0.455
Primary branches ΔNSC	0.322	0.490	0.329	0.797	1	0.811	0.420
Tertiary branches ΔNSC	0.720	0.650	0.552	0.902	0.811	1	0.629
Leaves ΔNSC	0.601	0.161	0.287	0.455	0.420	0.629	1
**P-value**	Medium roots ΔNSC	Coarse roots ΔNSC	Fine roots ΔNSC	Trunks ΔNSC	Primary branches ΔNSC	Tertiary branches ΔNSC	Leaves ΔNSC
Medium roots ΔNSC	0	0.319	0.071	0.075	0.308	0.008	0.039
Coarse roots ΔNSC	0.319	0	0.681	0.015	0.106	0.022	0.618
Fine roots ΔNSC	0.071	0.681	0	0.236	0.297	0.063	0.366
Trunks ΔNSC	0.075	0.015	0.236	0	0.002	0.000	0.138
Primary branches ΔNSC	0.308	0.106	0.297	0.002	0	0.001	0.175
Tertiary branches ΔNSC	0.008	0.022	0.063	0.000	0.001	0	0.028
Leaves ΔNSC	0.039	0.618	0.366	0.138	0.175	0.028	0

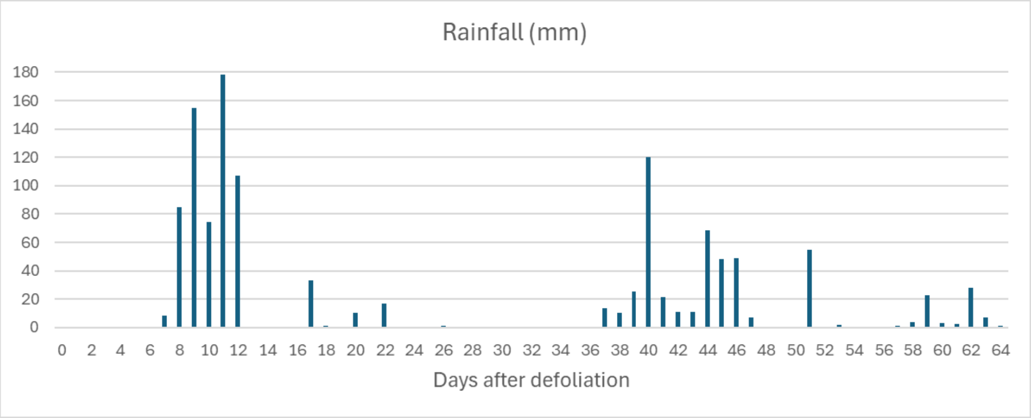

Supplementary figure S2: Daily rainfall between defoliation treatment implementation and harvest (2023-2024 growing season)*.

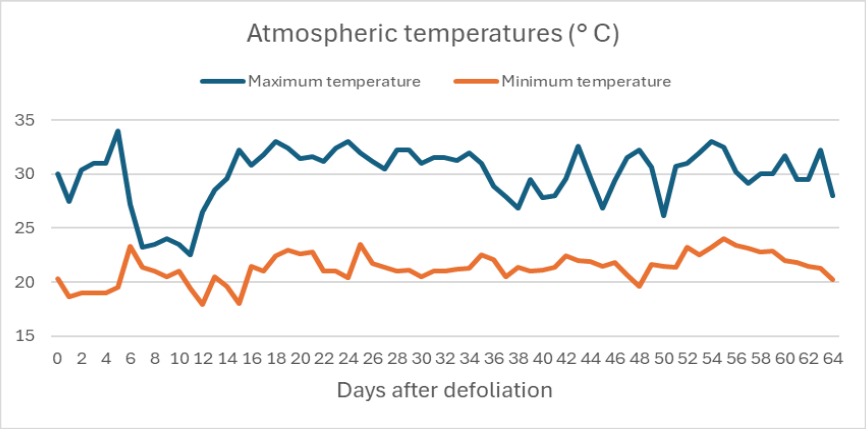

Supplementary figure S3: Daily minimum and maximum temperatures between defoliation treatment implementation and harvest (2023-2024 growing season)*.

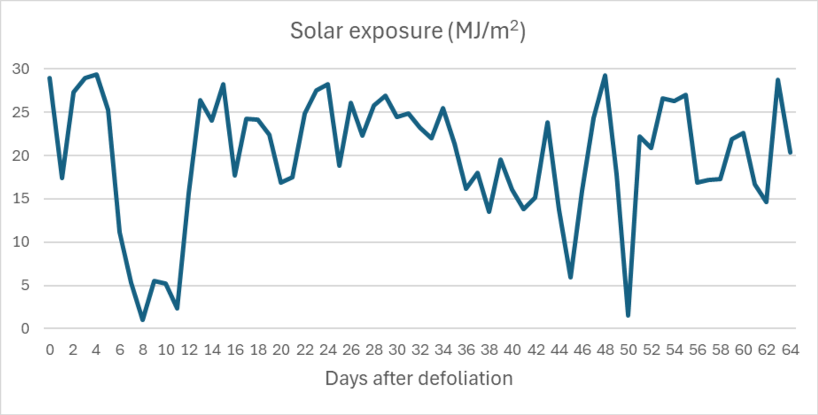

Supplementary figure S4: Daily solar radiation between defoliation treatment implementation and harvest (2023-2024 growing season)*.

* Meteorological conditions at Walkamin Research Station, obtained from Australian Bureau of Meteorology station ID 31108.
